# Stereochemistry‐Controlled Supramolecular Architectures of New Tetrahydroxy‐Functionalised Amphiphilic Carbocyanine Dyes

**DOI:** 10.1002/chem.201905745

**Published:** 2020-04-30

**Authors:** Boris Schade, Abhishek Kumar Singh, Virginia Wycisk, Jose Luis Cuellar‐Camacho, Hans von Berlepsch, Rainer Haag, Christoph Böttcher

**Affiliations:** ^1^ Forschungszentrum für Elektronenmikroskopie und Gerätezentrum BioSupraMol Institut für Chemie und Biochemie Freie Universität Berlin Fabeckstraße 36a 14195 Berlin Germany; ^2^ Institut für Chemie und Biochemie Organische Chemie Freie Universität Berlin Takustrasse 3 14195 Berlin Germany

**Keywords:** chirality, dyes/pigments, hydrogen bonds, supramolecular chemistry, synthesis design

## Abstract

The syntheses of novel amphiphilic 5,5′,6,6′‐tetrachlorobenzimidacarbocyanine (TBC) dye derivatives with aminopropanediol head groups, which only differ in stereochemistry (chiral enantiomers, *meso* form and conformer), are reported. For the achiral *meso* form, a new synthetic route towards asymmetric cyanine dyes was established. All compounds form J aggregates in water, the optical properties of which were characterised by means of spectroscopic methods. The supramolecular structure of the aggregates is investigated by means of cryo‐transmission electron microscopy, cryo‐electron tomography and AFM, revealing extended sheet‐like aggregates for chiral enantiomers and nanotubes for the mesomer, respectively, whereas the conformer forms predominately needle‐like crystals. The experiments demonstrate that the aggregation behaviour of compounds can be controlled solely by head group stereochemistry, which in the case of enantiomers enables the formation of extended hydrogen‐bond chains by the hydroxyl functionalities. In case of the achiral *meso* form, however, such chains turned out to be sterically excluded.

## Graphical abstract

**Sheets and tubes**: The syntheses of amphiphilic 5,5′,6,6′‐tetrachlorobenzimidacarbocyanine dye derivatives with aminopropanediol head groups, which only differ in stereochemistry (chiral enantiomers, *meso* form and conformer), are reported. The experiments demonstrate that the aggregation behaviour of compounds can be controlled solely by head group stereochemistry.

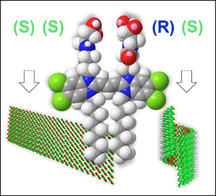

## Introduction

Cyanine dyes represent a class of organic dyes that are able to self‐assemble in polar solvents and on solid surfaces.^1^ Strong dipole–dipole coupling between the dye monomers leads to an electronically excited state shared by several monomers. Such exciton states cause a dramatic change in optical properties of the dye assemblies compared with the isolated molecules.^2^ Depending on the relative orientation between neighbouring monomers, different types of assemblies can be observed. A “head‐to‐tail” configuration of transition dipoles leads to a red‐shifted absorption band with large absorption cross section and super‐radiance.^2^ Such assemblies are called J or Scheibe aggregates after their discoverers, Jelly1a and Scheibe.1b The exceptional optical characteristics make J aggregates interesting tools for many applications, ranging from dye‐sensitised silver halide photography, light harvesting, photovoltaics and sensing to biomedical imaging.^3^

Aggregation in polar solvents is a common feature of dye molecules containing extended planar π‐electron systems. Further competing non‐covalent interactions, such as hydrogen bonding, halogen bonding or solvophobic forces, support the aggregation and control the formation of complex supramolecular structures. Because dipole–dipole coupling strength, and thus, photophysical properties of the aggregates are highly sensitive to the particular arrangement of the chromophores, controlled structuring of dye assemblies by tuning supramolecular interactions between individual building blocks facilitates the design of novel materials with desired properties. Powerful approaches in this regard are specific alterations of the chromophore side groups, incorporation of additives or modifications of the solvent polarity. In this context, a class of derivatives of the well‐known 5,5′,6,6′‐tetrachlorobenzimidacarbocyanine (TBC) chromophore^4^ have been investigated in detail by several groups.^5^ The chromophore was functionalised by attaching different polar or non‐polar substituents at the nitrogen atoms in the 1,1′‐ and 3,3′‐position. This allows for the tailored design of a large variety of supramolecular structures with interesting optical characteristics. By introducing 1,1′‐dioctyl substituents, Dähne and co‐workers advanced a class of amphiphilic dyes.5a

A fundamental prerequisite for studying dye assemblies is the availability of appropriate characterisation methods. Cryogenic transmission electron microscopy (cryo‐TEM) turned out to be an excellent technique to elucidate their morphology in the native environment of the solvent on the nano‐ to micrometre scale.^6^ Depending on the substituents of the TBC chromophore, one‐dimensional fibres, two‐dimensionally extended sheet‐like aggregates, and single‐ or multi‐layered tubular architectures with a helical molecular organisation were detected. The last of these became a matter of particular interest because the shape and size of such tubular aggregates resemble the natural light‐harvesting system in green sulfur bacteria.^7^ Thorough optical characterisations by means of linear optical spectroscopy, usefully supplemented by nonlinear techniques^8^ or theoretical structure modelling,5e, 9 are mandatory for a prospective use of photophysical characteristics. Although the understanding of dye assemblies has made excellent progress in recent years, a substantial challenge remains: how does a particular molecular modification control the morphological, and thus, optical characteristics of the final supramolecular structure? So far, empirical approaches are still necessary due to a missing conclusive concept.

The most extensively investigated representatives of tube‐forming TBC‐based cyanine dyes are amphiphilic C8O3, bearing two carboxylic acid groups, and C8S3, bearing two sulfonic acid groups.5a, 10 (For abbreviations of TBC‐based dyes, see Table 1.) In aqueous media, they are converted into their conjugate bases, yielding negatively charged aggregate surfaces. Moreover, previous studies revealed that the helicity of C8O3 tubes could be tuned upon the addition of chiral alcohols.^11^

**Table 1:** Abbreviations for TBC‐based dyes discussed herein.

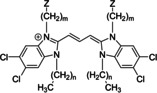			
	Z	*n*	*m*
TDBC (C2O4)	−SO_3_	1	4
C8S3	−SO_3_	7	3
C8O3	−COOH	7	3

The goal of the present study was to prevent surface charge effects and to investigate the impact of chiral head groups on the supramolecular architecture of assemblies.

**Scheme 1:**
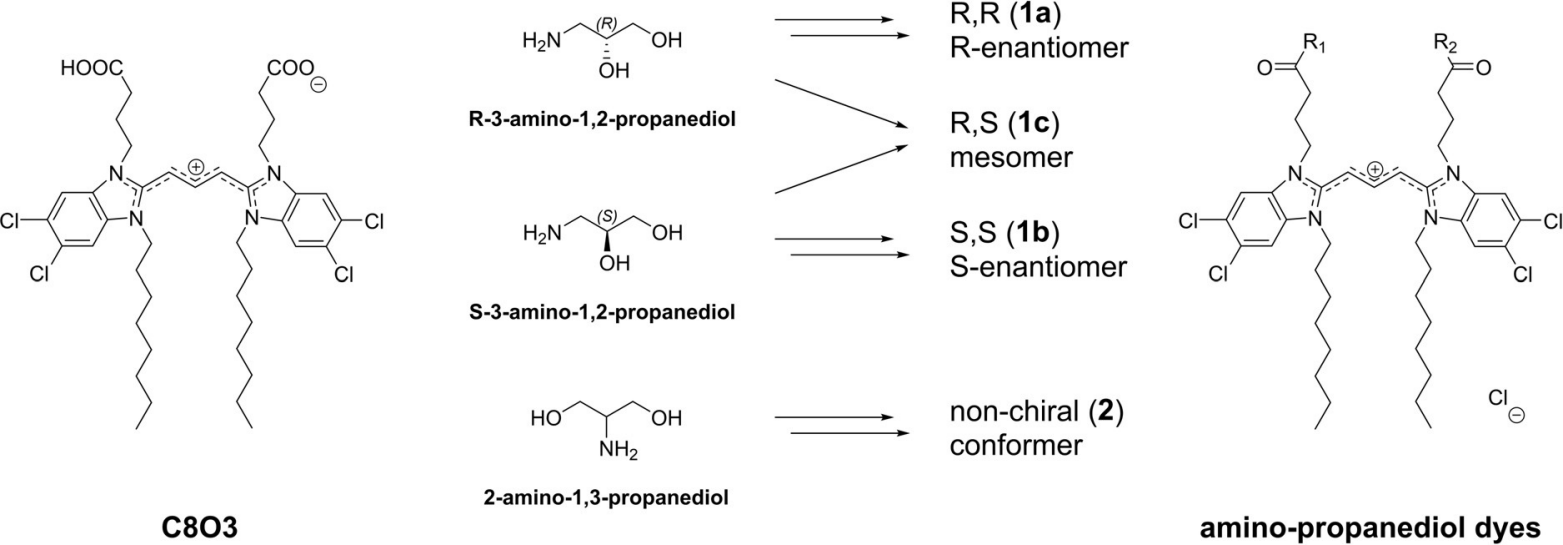
Chemical structure of the parent dye C8O3 and the family of newly synthesised aminopropanediol isomers.

To achieve this goal, we selected C8O3 as the parent TBC derivative and functionalised both its carboxyl groups with aminopropanediol, which provided a well‐balanced amphiphilic character to allow for the formation of aggregates. Since there are two conformations of aminopropanediol, chiral 1‐amino‐2,3‐propanediol and non‐chiral 2‐amino‐1,3‐propanediol, amidation of the two symmetrically situated carboxyl groups in C8O3 renders the formation of four different isomers possible, namely, two enantiomers with either *R*,*R* or *S*,*S* configuration, a *meso* form with *R*,*S* configuration and the non‐chiral conformer derived from 1‐amino‐2,3‐propanediol (Scheme 1). Hence, our approach allowed for versatile molecular alterations, while the hydrophilic–lipophilic balance (HLB) of the dyes and the spatial demand of the head groups remained unchanged. Moreover, it enables studies on the impact of the molecular chirality. With this structural diversity, we were able to elucidate the specific influence of head group stereochemistry and/or conformation on the aggregation behaviour of the novel amphiphilic cyanine dyes.

Studies of the C8O3 and C8S3 derivatives were used as guidelines for the present investigations and provided the basis for comparisons. The new derivatives were first characterised as monomers in organic solvents by means of absorption and fluorescence spectroscopy. Subsequently, aggregation in pure water was followed by absorption, circular dichroism (CD), linear dichroism (LD), and fluorescence spectroscopy and structurally monitored by cryo‐TEM, cryogenic electron tomography (cryo‐ET) and AFM. Structure models are presented and discussed.

## Results and Discussion

### Synthesis

**Scheme 2:**
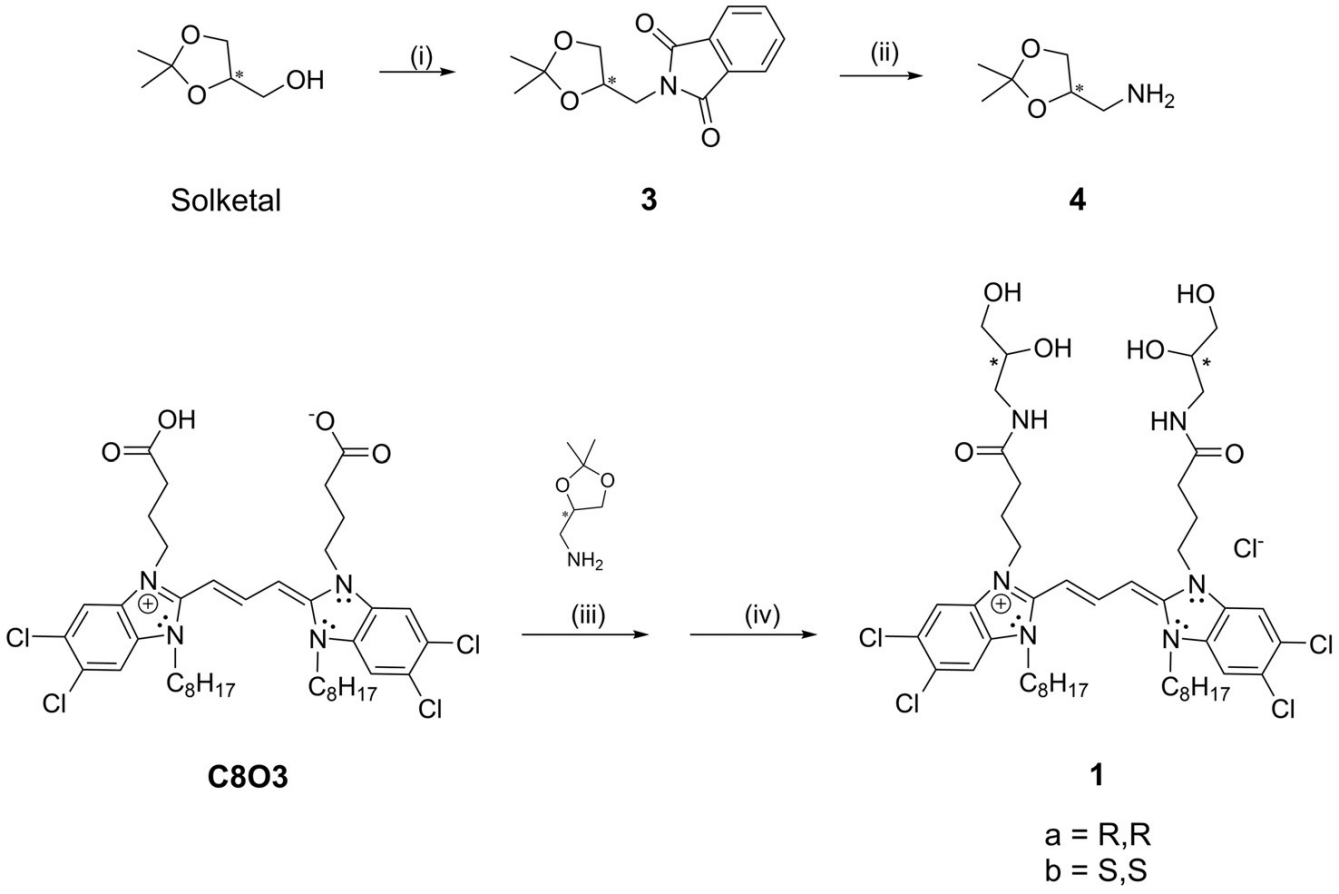
Synthesis of cyanine dye tetrahydroxy derivatives. i) PPh_3_, phthalimide, diethyl azodicarboxylate (DEAD), THF, RT, 20 h; ii) NH_2_−NH_2_, H_2_O, MeOH, reflux, 4–5 h; iii) 1‐[bis(dimethylamino)methylene]‐1*H*‐1,2,3‐triazolo[4,5‐*b*]pyridinium 3‐oxide hexafluorophosphate (HATU), *N*,*N*‐diisopropylethylamine (DIPEA), DMF, RT, 2 h; iv) HCl, MeOH, RT, 2–5 h.

The synthesis of aminopropanediol cyanine dyes (compounds **1 a**–**c** and **2**) has been achieved by two different synthetic approaches. A straightforward synthetic route was followed to obtain isomers with uniform head groups, that is, the *R*,*R* or *S*,*S* enantiomers from 3‐amino‐1,2‐propanediol (Scheme 2), and the non‐chiral symmetric serinol derivative (Scheme S4 in the Supporting Information). To achieve and preserve the desired stereochemistry and conformation of the chiral head groups, commercially available enantiopure solketal, that is, the acetal of 1,2,3‐propane diol, was chosen as a starting material. This solketal (*R* or *S*) was converted into the respective solketal amine **4** in two steps (Scheme 2) and was then coupled to the acid groups of C8O3, followed by deprotection of the acetal groups under mild acidic conditions. The achiral serinol derivative **2** was synthesised accordingly by using commercially available 2‐amino‐1,3‐propanediol (serinol) without protection of the OH groups, following the same coupling procedure (Schemes S1–S3 in the Supporting Information).

**Scheme 3:**
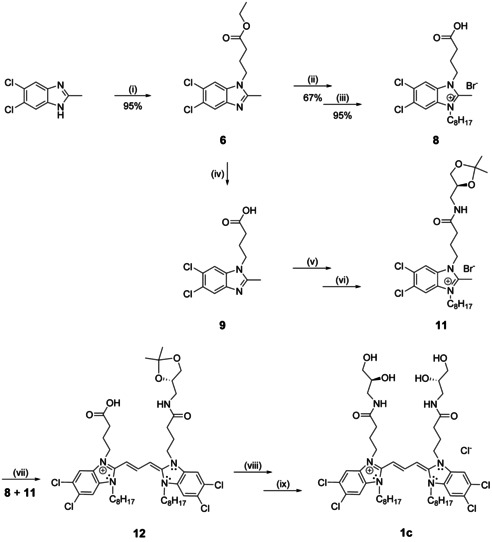
Synthesis of asymmetrical benzimidazoles. i) Ethyl bromoacetate, NaOH, DMSO, RT, 48 h; ii) 1‐bromodecane, 150 °C, 6 h; iii) HBr (48 %), water, 120 °C; iv) KOH, ethanol, reflux, 12 h; v) *N*‐(3‐dimethylaminopropyl)‐*N*′‐ethylcarbodiimide hydrochloride (EDC**⋅**HCl), 4‐dimethylaminopyridine (DMAP), DMF, 24 h, RT; vi) 1‐bromodecane, 150 °C, 6 h; vii) 1,8‐diazabicyclo[5.4.0]undec‐7‐ene (DBU), CH_3_I, methanol, RT, 48 h; viii) (*R*)‐solketal, EDC**⋅**HCl, DMAP, DMF, 24 h, RT; ix) HCl, methanol, 6 h, RT. (see the complete synthetic scheme in the Supporting Information).

Despite the convenient synthetic route outlined above, a different strategy was necessary to synthesise the enantiopure *meso* form (**1 c**; Scheme 3). This mirror‐symmetrical dye derivative was synthesised from two different monomers to attain its specific *R*,*S* conformation selectively. For that, 5,6‐dichloro‐2‐methylbenzimidazole was subsequently treated with ethyl 4‐bromobutanoate and 1‐bromooctane followed by hydrolysis in the presence of aqueous HBr to yield benzimidazole **8**. Benzimidazole **11** was obtained by ester hydrolysis of **6**, amidation with (*S*)‐solketal amine, and finally N‐alkylation with 1‐bromooctane (Scheme 3).

Both benzimidazoles **8** and **11** were coupled in methanolic solution in the presence of DBU and CH_3_I. As expected, three different combinations of these two monomers led to the formation of three different dyes (Scheme S3 in the Supporting Information). From their polarity order, however, it was possible to extract the desired cyanine dye **12** by performing column chromatography. Amidation with (*R*)‐solketal amine and subsequent acetal deprotection finally yielded the mesomer (**1 c**).

### Spectroscopic characterisation

**Figure 1:**
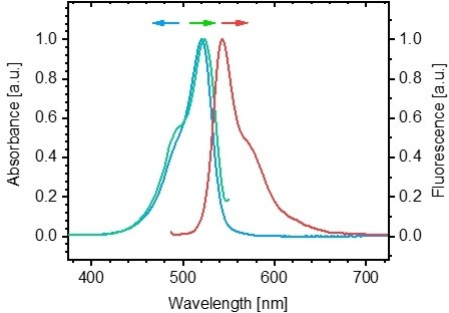
Normalised absorption (blue) and fluorescence spectra, excitation (green) and emission (red) of the new aminopropanediol *R* enantiomer (**1 a**) in methanol. The spectra are representative for all four compounds **1 a**–**c** and **2**. Concentrations are 0.1 mm for UV and 0.01 mm for fluorescence measurements. The excitation spectrum was collected at *λ*=550 nm; the emission spectrum was collected after excitation at *λ*=480 nm.

The aminopropanediol dyes (**1 a**–**c** and **2**) are readily soluble in MeOH and DMSO. In MeOH, they all show an identical absorption band with a maximum at *λ*=520 nm (full‐width at half‐maximum (fwhm)=932 cm^−1^) and a vibronic shoulder at *λ*≈485 nm (see Figure 1 for the *R* enantiomer (**1 a**)). The fluorescence emission spectrum reveals a mirror image of the absorption band with a maximum at *λ*=544 nm, yielding a Stokes shift of 24 nm. The corresponding excitation spectrum resembles the absorption spectrum. The spectra are in good agreement with those of the parent derivative C8O3, indicating that the introduction of the non‐ionic aminopropanediol head groups with their particular chirality does not affect the spectroscopic properties of the chromophore. The spectroscopic features are typical for cyanine dye monomers.^12^

**Figure 2:**
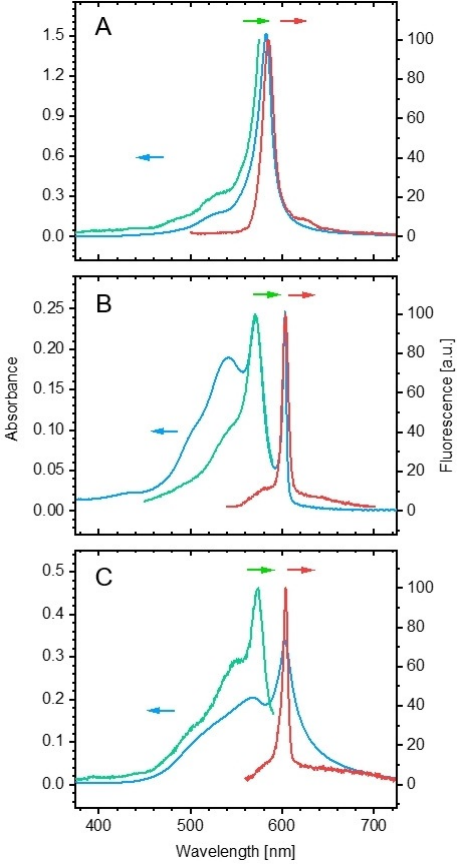
Absorption (blue) and fluorescence spectra, excitation (green) and emission (red) of A) the *R* enantiomer (**1 a**), B) the mesomer (**1 c**) and C) the conformer (**2**) in water. Matured stock solutions were diluted to concentrations of 0.1 mm for UV/Vis and 0.01 mm for fluorescence immediately prior to measurements. The fluorescence excitation spectra were collected at A) *λ*=527, B) 604 and C) 604 nm and the emission spectra were collected after excitation at A) *λ*=488, B) 530 and C) 550 nm, respectively.

The spectra of the dyes in water differ significantly from the monomer spectra in organic solvents (Figure 2). Remarkable bathochromic shifts of the absorption band indicate the formation of J aggregates1c in all cases (**1 a**–**c**, **2**), and the influence of the chirality of the compounds becomes clearly visible by the differing absorption spectra of the enantiomers (**1 a** and **1 b**) and *meso* form (**1 c**).

Spectra of both enantiomers (**1 a** and **1 b**) are identical. The absorbance is characterised by a single narrow band at *λ*=583 nm (fwhm=413 cm^−1^) with a shoulder at *λ*=535 nm (shown for the *R* enantiomer (**1 a**) in Figure 2 A as an example). The fluorescence emission has its maximum at *λ*=585 nm. This nearly resonant emission is another typical feature of J aggregates.1c The related excitation spectrum is in good agreement with the absorption spectrum. With respect to their shape, all of these spectra are similar to those found for the J aggregates of the closely related cyanine dyes TDBC (C2S4)5b and C8O4.^13^

In contrast, the mesomer (**1 c**) shows a split absorption spectrum (Figure 2 B) with a sharp and intense sub‐band at *λ*=603 nm (fwhm=165 cm^−1^) and a second narrow and intense sub‐band at *λ*=572 nm; a third broader band of lower intensity is located at *λ*≈543 nm. The emission spectrum shows only one sharp band in resonance with the absorption band at *λ*=603 nm (fwhm=247 cm^−1^), regardless of the excitation wavelength.

**Figure 3:**
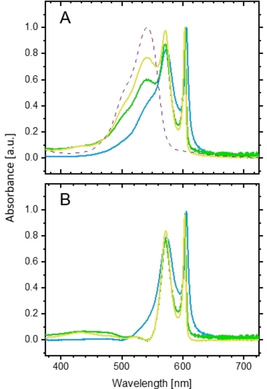
Three individual preparations from three different batches of the mesomer **1 c** in Milli‐Q water (yellow, green and blue) show different strengths of the third sub‐band at *λ*≈543 nm, whereas the two longer wavelength bands remain almost stable (A). The individual spectra are normalised to the same absorbance at *λ*≈603 nm. The dotted spectrum was derived from a solution of **1 c** in HCl at pH<4. After appropriate scaling and subtraction of this low‐pH spectrum from the three spectra in Milli‐Q water, two‐banded spectra are obtained for all preparations (B).

Figure 3 A shows a family of spectra from three individual mesomer sample preparations of **1 c**. The comparison reveals differences in the intensity of the third absorption band at *λ*=543 nm, which clearly exceeds experimental error limits. Also, the maximum position of the longest wavelength band varies slightly from sample to sample, but to a much lesser extent (between *λ*=603 and 606 nm). It is noticeable, however, that the intensity ratio of the first and second sub‐bands remains almost constant. If the mesomer was dissolved in aqueous HCl at low pH (lower than 4), the solution displayed a spectrum with a maximum at *λ*=543 nm (purple dotted line), which resembled that of monomers (Figure 1), but was clearly broader (fwhm=1295 cm^−1^) and red‐shifted by about 23 nm. After appropriate scaling and subtraction of this band from the spectra in Milli‐Q water (Figure 3 A), roughly identical, two‐banded spectra are obtained for all three preparations (Figure 3 B). This simple fit procedure suggests that the remaining longer wavelength bands belong to the aggregate, whereas the variable third band at *λ*≈543 nm might indicate the presence of a further independent species (probably dimers or smaller oligomers).

The conformer (**2**) displays a split absorption spectrum in water (Figure 2 C, blue), as described for **1 c**. In the case of **2**, the bands became broader and their intensity weakens within a period of minutes, which indicates precipitation. The fluorescence behaviour, however, is similar to that of the mesomer. Due to rapid precipitation, the conformer was not investigated in more detail by means of spectroscopy.

**Figure 4:**
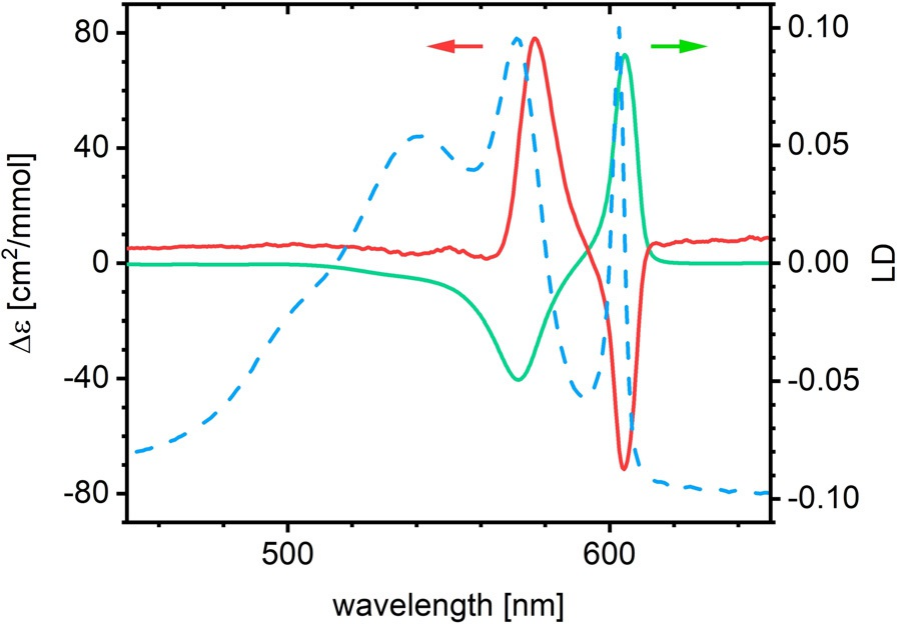
CD (red line) and LD (green line) spectra of an aqueous solution of the achiral mesomer **1 c**. To compare the band positions, a normalised isotropic absorption spectrum (blue broken line) is added to the graph without a dedicated ordinate.

In previous studies, we demonstrated that the achiral parent dye C8O3 formed optically active helical J aggregates by applying CD spectroscopy and cryo‐TEM. Moreover, we were able to tune the handedness of aggregates by adding chiral alcohols.10a, 11 Therefore, we were interested to see whether the use of chiral head groups affected the supramolecular assembly in a similar way. The absence of CD signals from methanolic solutions of the dyes indicates that the chirality of head groups has, at best, a negligible effect on the conformation of the monomeric (non‐aggregated) chromophores.^14^ In contrast, the aggregated mesomer (**1 c**) shows negative and positive Cotton effects at *λ*=577 and 605 nm, respectively (Figure 4, red line). Phenomenologically, this behaviour is in line with corresponding CD results of the parent C8O3 dye and indicates a similar molecular architecture of aggregates. For this dye, a tubular architecture was detected, with the tube wall being composed of two helically twisted chromophore layers.5b The observed Cotton effects were ascribed to molecular excitons. In the case of the related C8S3 dye, the helical architecture of tubes was even proven directly from highly resolved cryo‐TEM images.^6^, ^15^ For the enantiomers (**1 a** and **1 b**), however, we did not obtain spectra with mirror symmetry, which indicated the absence of a chiral supramolecular organisation. Morphological investigations proved this claim (see below).

LD spectroscopy is a highly sensitive method that can give valuable information about the molecular packing orientation within aggregates. Isotropically oriented molecules give no LD signal, whereas anisotropic aggregates with a high aspect ratio give characteristic LD signals. To measure the LD, the aggregates dispersed in the sample have to be aligned.^16^ For fibrous aggregates (cf. Figures 8 and 9, below), this can simply be accomplished in the streaming field of a Couette flow cell. Solutions of **1 c** show LD spectra (Figure 4, green line) consisting of two strong sub‐bands, the locations of which coincide with the two strongest absorption bands. The positive LD signal of the longest wavelength band (*λ*=604 nm) indicates a parallel polarisation of the associated transition with respect to the long axis of the aggregates. The other band (*λ*=571 nm) is polarised perpendicularly. This polarisation pattern is characteristic for single‐layered tubular J aggregates.5e, 17

Analog LD measurements of the enantiomer solutions gave only one small positive band. This finding is difficult to interpret because the sheet‐like structure of these aggregates (cf. Figure 6, below, as evidence) casts serious doubts on their ability to be directionally oriented in the Couette cell.

Due to the solubility of dyes in methanol, disaggregation can simply be monitored by means of absorption spectroscopy upon methanol titration. Such measurements provide additional information about the stability of aggregates and the kinetics of disaggregation. In the experiments reported herein, we added a methanolic dye solution to the respective aqueous solution of the aggregated dyes to keep the dye concentration constant.

**Figure 5:**
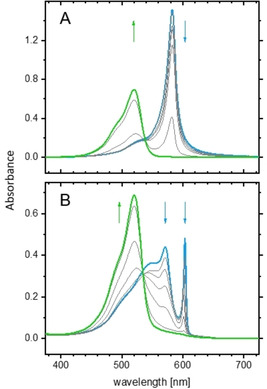
Disaggregation of J aggregates of A) the *R* enantiomer **1 a** and B) the mesomer **1 c** upon MeOH titration. The equilibration time between each titration step was 5 min. Starting solution: 0.1 mm dye in pure water. Arrows indicate the effect of increasing MeOH concentration: A) [MeOH]: 0, 10, 20, 30, 40, 50 and 60 %; B) [MeOH]: 0, 10, 20, 30, 40, 45, 50 and 60 %.

Figure 5 displays sets of absorption spectra for the *R* enantiomer (**1 a**; Figure 5 A) and the mesomer (**1 c**; Figure 5 B). The absorbance of **1 a** remains almost unchanged in an admixture of up to 30 vol % methanol. Further increasing the methanol content to 60 vol % promotes the gradual formation of monomers at the expense of J aggregates. A defined isosbestic point at *λ*=540 nm indicates disassembly of J aggregates directly into monomers without the appearance of intermediates.

A slightly different result was obtained for the mesomer (Figure 5 B). Here, the isosbestic point is located at *λ*=535 nm. In contrast to the *R* enantiomer, the J bands remain almost unchanged up to 20 vol % methanol. Complete disassembly of the aggregates is accomplished at 50 vol % methanol. Noticeable is the appearance of a hump at *λ*≈540 nm prior to complete disassembly. By deconvolution of the spectra into monomer and aggregate components (not shown), a corresponding band could indeed be extracted, but an associated aggregate species could not be detected by means of cryo‐TEM.

### Structural characterisation

Spectroscopic investigations of all aqueous solutions of aminopropanediol dyes (**1 a**–**c**, **2**) were complemented by direct structural characterisation through cryo‐TEM, cryo‐ET and AFM imaging techniques.

#### Sheet‐like assemblies of the enantiomers

**Figure 6:**
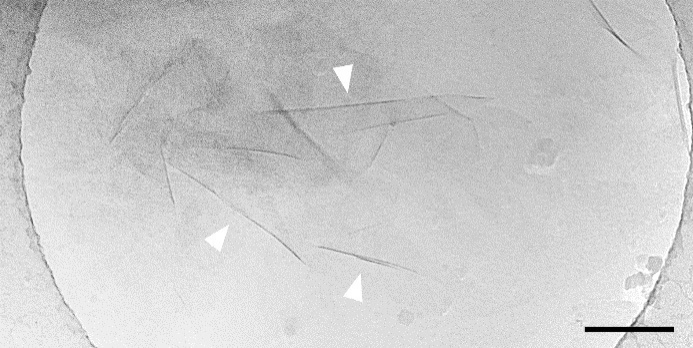
The cryo‐TEM image of a 0.1 mm solution of the *R*,*R* enantiomer **1 a** in pure water reveals the formation of sheet‐like aggregates. Their approximate thickness can be roughly estimated from wrinkles (arrowheads) to measure (6±1) nm. Scale bar: 200 nm.

Both enantiomers (**1 a** and **1 b**) assemble into sheets with dimensions in the micrometre range (Figure 6). The sheets are separated without any tendency to form stacks. Occasionally emerging wrinkles (dark lines marked by white arrowheads) were used to estimate a sheet thickness of approximately 6 nm. This value, however, is only a rough estimate due to inhomogeneous folding events and blurring at the edges of the folds.

**Figure 7:**
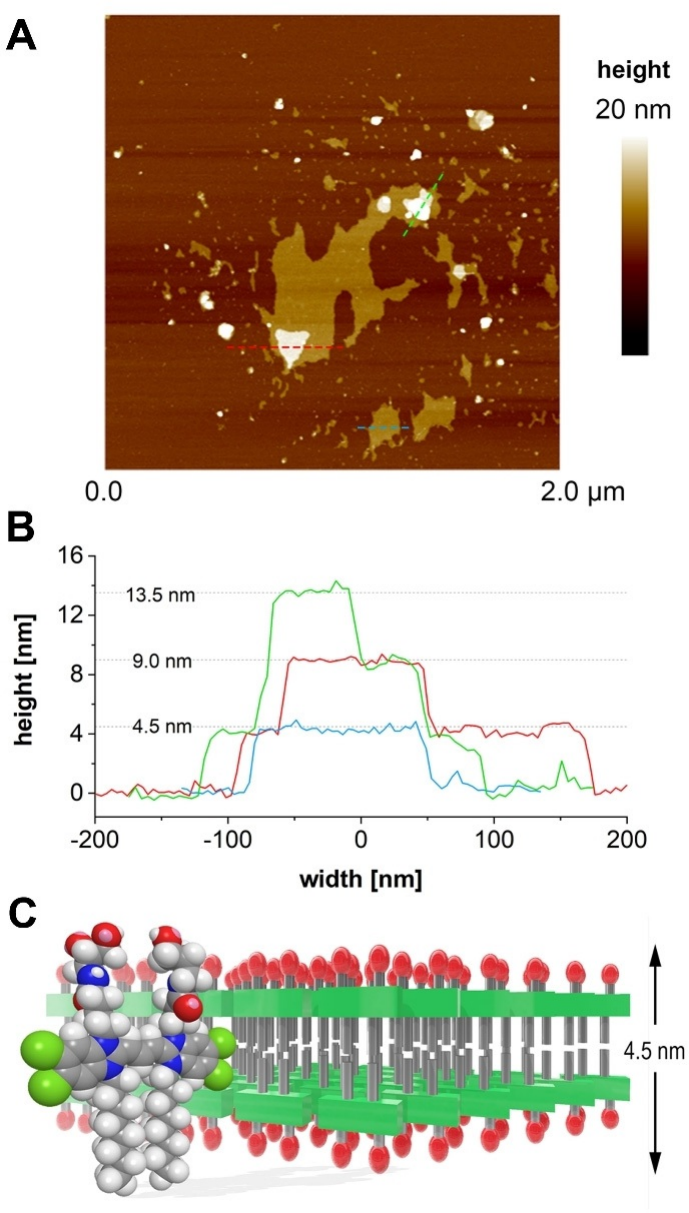
A) AFM image revealing the deposition of sheet‐like aggregates from the *R* enantiomer **1 a** on mica. B) The height profiles measured along the trajectories depicted by dashed lines in the top‐view image show recurring 4.5 nm steps, even if smaller aggregates are deposited on top of the large sheets (red and green lines). C) Space‐filling model of the aminopropanediol cyanine dye and a schematic representation of a J‐type bilayer arrangement thereof. The model reproduces the distance between opposing chromophores and the overall thickness of the sheet‐like aggregates from the enantiomers, as elucidated from the AFM measurements. TBC chromophores in a brickwork arrangement are represented by green blocks, diol head groups by red ovals and hydrocarbon chains by grey rods.

For a more accurate determination of the thickness, AFM measurements were exemplarily performed on *R*‐enantiomer sheets deposited on mica support in a water‐filled liquid chamber. Sheets with a thickness of 4–4.5 nm were imaged in PeakForce mode (Figure 7 A, B). Even smaller sheets deposited on top provided additional plateaus of about 4.5 nm high. The value corresponds approximately to twice the length of the molecule, and thus, indicates a bilayer arrangement of dye molecules (Figure 7 C). Despite a high level of order, as suggested by the narrow absorption band (cf. Figure 2), ultra‐structuring of the surface of sheets could not yet be resolved.

#### Tubular assemblies of the mesomer

Other than the enantiomers, the mesomer (**1 c**) forms tubular supramolecular aggregates. In one particular preparation, we found almost exclusively individual tubes with maximum lengths reaching several micrometres, whereas other preparations predominantly showed tube bundles with varying degrees of twist. Once formed, the particular proportions of tubes and bundles in either sample persisted over time (Figure S2 in the Supporting Information). The heterogeneity in preparation‐dependant structural varieties, however, remains unexplained.

**Figure 8:**
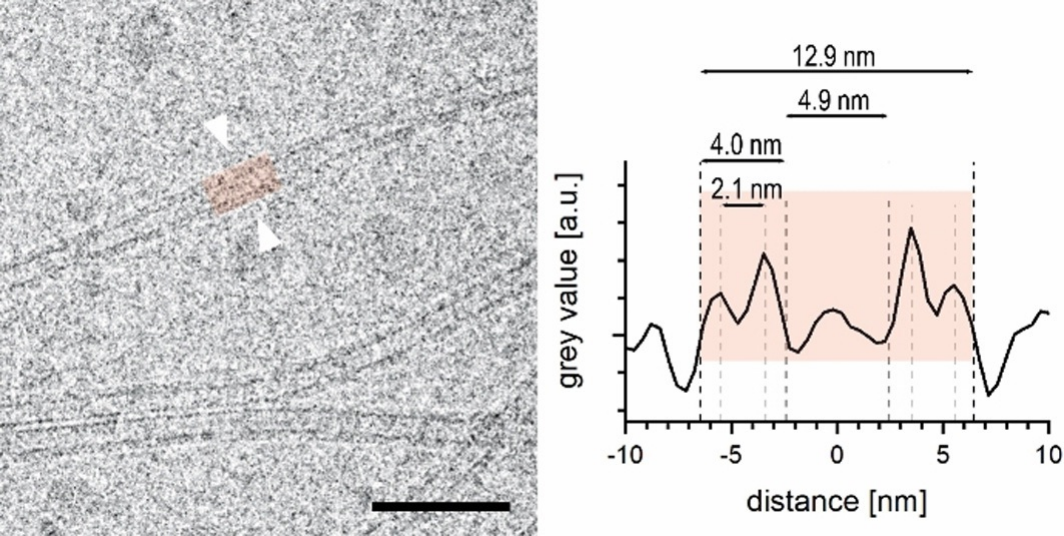
Left) The cryo‐TEM image of a two‐day‐old 1 mm solution of the mesomer **1 c** in pure water displays uniform individual tubes. Their lengths often exceed several micrometres. The line plot (right) across the highlighted tube section provides the cross‐sectional density, and thus, allows for the precise characterisation of the double‐layer structure of the tubes and their dimensions. Scale bar: 50 nm.

Figure 8 displays a sample of **1 c** in which almost exclusively individual tubes were formed. The high spatial image resolution reveals the double‐layer architecture of the tube walls. The line plot (right) averages the grey values of a 41 nm long straight tube section (red area) along its central axis, and thus, provides the cross‐sectional electron density with enhanced signal‐to‐noise ratio of the tubes. The labelled tube has an outer diameter of about 12.9 nm and an inner diameter of about 4.9 nm. The total thickness of the wall is about 4.0 nm (dark dashed lines) and the peak to peak distance (highest densities) between the layers measures 2.1 nm (light dashed lines) and corresponds to previous data of C8O3 tubes.5b, 18 The peaks mark the positions of the electron‐rich dye skeleton (chromophore). Thus, the profile of the bent double layer of the tubes is in good agreement with that of the flat double layer (cf. Figure 7 C) and matches the molecular dimensions.

To look for long‐term changes in morphology, we re‐examined the 1 mm solution after 2, 21 and 125 days. Statistical analysis of a total of 344 line plots (Figure 8), that is, approximately 100 from each sample, yielded mean diameters of 12.7, 12.5 and 12.7 nm, with standard deviations of 0.76, 0.97 and 0.64 nm, respectively, for each subset. This proves a robust geometry of the individual tubes, even upon long‐term storage.

The (outer) tube diameter is a little larger than that of the parent dye C8O3 (for pure dye: ≈10.0 nm,5b for poly(vinyl alcohol)‐stabilised tubes: 11.5 nm^18^) and comparable with that of the sulfonic acid analogue, C8S3, prepared by the alcoholic route (13 nm9a, 19). The wall thickness of about 4 nm, however, is almost identical for all tubes.

**Figure 9:**
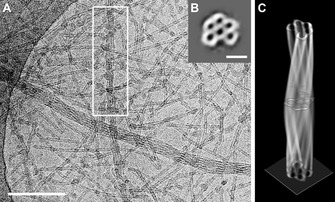
A) Cryo‐TEM image of the mesomer **1 c** in a 1 mm aqueous solution, which was subjected to cryo‐ET, showing individual tubes and twisted tube bundles. Scale bar: 200 nm. B) A summed image of aligned slices extracted perpendicular to the long axis of the labelled bundle from the reconstructed volume provides a mean cross‐sectional density with enhanced signal‐to‐noise ratio (note the inverted contrast necessary for image processing). Due to rotation of the motif upon following the long axis of the bundles, the missing wedge artefact of the tomography reconstruction can be overcome. Scale bar: 50 nm. C) The averaged cross section was used to reconstruct a 3D model of the bundle, which is presented in voltex^20^ representation to illustrate the twisted course of the constituent tubes.

Figure 9 A displays a cryo‐TEM image of a 1 mm solution of the mesomer (**1 c**). Here, next to short individual tubes tube bundles, such as those often found for the parent dyes C8O35b, 21 and C8S3,5e, 19 are also obtained.

To elucidate the three‐dimensional volume structure of the twisted bundles, we subjected the sample to cryo‐ET. The tomogram was reconstructed from 65 single exposures taken at angles from −65 to 63° in 2° tilt increments. Slices of the tomogram along the boxed bundle (Figure 9 A) provide five tubes that are hexagonally arranged around a sixth central tube (Figure 9 B). This composition leaves a void at the outer face of the aggregate, which permits the continuous displacement of this pattern to be followed along the long axis of the bundle by scrolling through slices of the tomogram (see Figure S4 in the Supporting Information). A complete 360° cycle, corresponding to the pitch of the bundle, is 400 nm. A total of 232 extracted slices were used to calculate the mean cross section of the bundle with an enhanced signal‐to‐noise ratio (Figure 9 B). Due to the complete 360° rotation of the motif along the long axis of the bundle, a complete and accurate density profile is averaged without restriction of the missing wedge artefact. This motif was used for the reconstruction of the complete volume of the bundle (Figure 9 C). It becomes apparent that the wall thickness between the tubes and towards the exterior of the bundle is the same, which reveals that the double‐layer construction of the tube walls is absent in the interior of the bundles, where tubes should form two double layers at their contact faces.

Recently, Eisele et al. elucidated the same deviation of tube layers for bundles of C8S3 by using cryo‐ET volume reconstructions.5e They assumed that, upon bundling, the originally bilayered tubes lose their outer layer. In the following, we describe an image‐based approach to reveal an even more detailed insight into this unusual molecular tube layer organisation.

#### Modelling of tube bundles

Due to the limited resolution in cryo‐ET data, more detailed information about the supramolecular construction can be obtained by analysing individual cryo‐TEM projection images recorded at higher magnification by a geometry‐based simulation approach. We were particularly interested in the structural organisation at the interfaces of adjacent tubes. Hereby, the hexagonal arrangement of tubes, as observed by cryo‐ET (cf. Figure 9 B), provided the basic motif.

**Figure 10:**
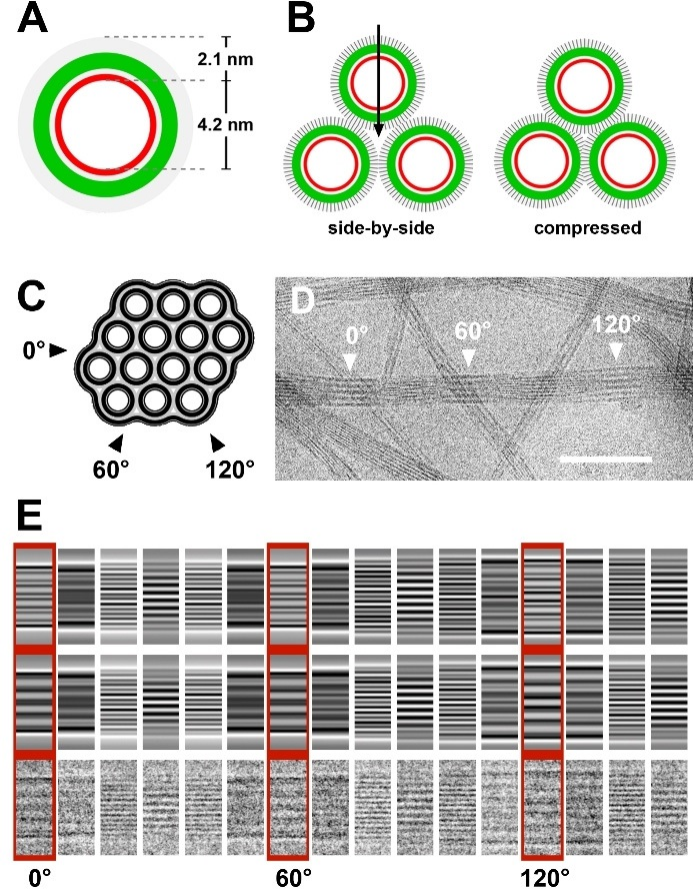
Modelling of a twisted tube bundle. A) Cross‐sectional view of a model tube with a monolayered wall. Constituent dye molecules are oriented with their head groups (red) towards a central channel, whereas octyl chains (grey) point outwards. B) Packing motifs for tubes in a bundle. Side‐by‐side arrangement of the tubes (left), leaving voids at the interfaces. Compressed packing of the tubes (right) diminishes interspaces. C) Cross section of a bundle of monolayered tubes (see A), reproducing the number and orientation of the tubes in the original bundle in D). This cross section was used to generate a 3D model of the respective bundle (cf. Figure S6 in the Supporting Information). D) Cryo‐TEM image of a twisted bundle with the marked positions of characteristic line patterns. E) Back projection patterns in 10° steps of 3D models calculated for the side‐by‐side arrangement (row 1), the compressed packing (row 2) and the experimental cryo‐TEM data (row 3). Projection images of the compressed arrangement reveal a much better match with the experimental data.

The projection pattern along the twisted bundle in Figure 10 D was used as an experimental reference. Because the changes in the pattern along the bundle axis are a structural equivalent of rotation around the bundle axis (as proven by the cryo‐ET data), we compare the systematic variation in the experimental projection pattern with that of a rotated hexagonally packed bundle of tubes, the multiplicity and tube diameter of which can be determined directly from the cryo‐TEM image (cf. Figure S5 in the Supporting Information). For the case at hand (see Figure 10 D), we determined multiplicities of 4, 4 and 5 layers in the directions of 0, 60 and 120°, respectively, and a tube diameter of 8.4 nm from line distances at 30, 90 or 150°. These values indicate a tube arrangement, as shown in Figure 10 C.

We calculated the back‐projections of the respective 3D volume and compared them with the experimental data. The back‐projection patterns of the side‐by‐side arranged tube bundle (Figure 10 E, row 1) coincide fairly well with the data. A compressed (interdigitated) arrangement of monolayered tubes (Figure 10 B, right), however, revealed a much better fit (Figure 10 E, row 2). Moreover, in this arrangement, unfavourable voids at the trigonal contact interfaces of the tubes (arrow in Figure 10 B, left) are largely diminished (right).

**Figure 11:**
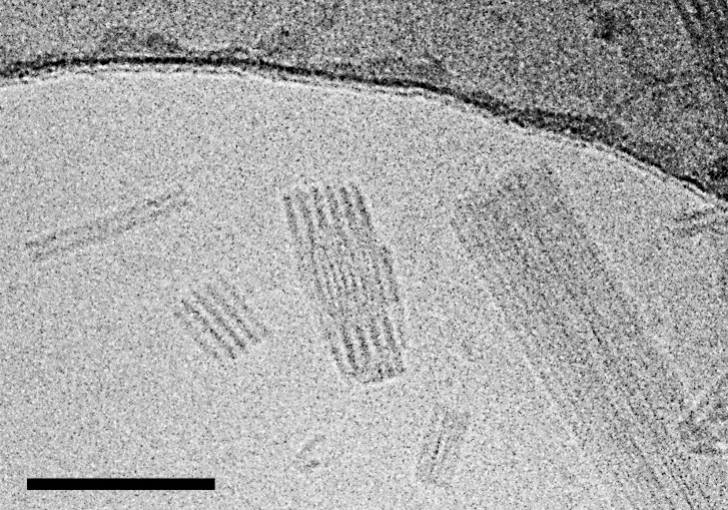
Twisting and bundling starts at the early state of the aggregation process. This micrograph shows a vitrified sample prepared 6 h after the dissolution of solid matter in water. Next to short individual tubes, a short (≈100 nm length) twisted bundle can be found. Scale bar: 100 nm.

To obtain information about the assembly process for the tube bundles, we prepared early states of aggregation. A cryo‐preparation 6 h after dissolution of the dye shows short, only 50–100 nm long, bundles (Figure 11), which already exhibit the typical patterns of a twisted bundle (cf. Figure 9). Its existence at this early stage of the aggregation process, as well as the persistence of individual tubes, indicates a new aspect of assembly growth, that is, the hierarchical organisation of tubes into bundles clearly results from intrinsic supramolecular interactions at the start of aggregation and contravenes the assumption that bundling and twisting is caused in a multi‐stage process over time.

#### Needle‐shaped crystals of the conformer

The conformer (**2**) showed macroscopically visible needle‐like aggregates that were too large to prepare for cryo‐TEM. Upon addition of methanol, however, the needles became significantly smaller, and thus, enabled cryo‐TEM investigations.

**Figure 12:**
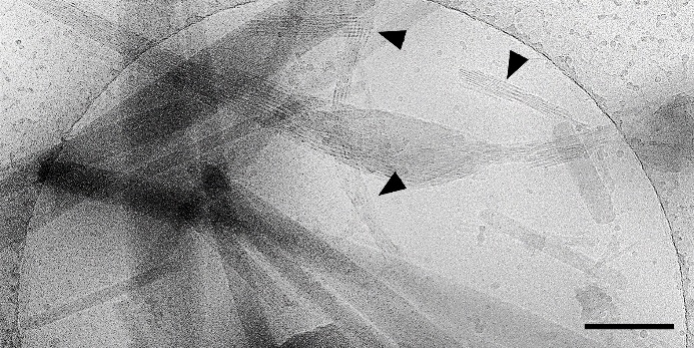
Cryo‐TEM image of an aqueous solution of the conformer **2**, containing 30 vol % of methanol, displaying a few tube bundles (black arrowheads) and predominantly wide, elongated and non‐twisted crystalline assemblies. Scale bar: 100 nm.

In contrast to **1 c**, cryo‐TEM images of aqueous solutions of **2** revealed very few individual tubes or tube bundles (Figure 12, black arrowheads); predominantly, the formation of smooth, elongated structures occurs. The latter can reach lengths of several micrometres and widths of hundreds of nanometres. Despite their dimensions, the large assemblies seem to preserve some flexibility because many of them are slightly bent. Nevertheless, frequently occurring very narrow line patterns suggest a repetitive crystalline order. These morphological findings are in line with the observed spontaneous precipitation of dye **2** and the remarkably broad absorption bands. Although tube‐like aggregates were formed in negligible amounts, crystallisation seems to dominate over supramolecular aggregation in case of **2**.

## Discussion

Monomer spectra of all novel dyes coincide with the respective data of the parent dye C8O3, which proves that modifications in the head group chemistry have no effect on the electronic properties of the chromophore. In water, red‐shifted absorption spectra indicate the formation of J aggregates for all four dyes. Spectral differences among the new dyes, as well as in relation to C8O3 or C8S3, which only differ in hydrophilic head groups, indicate different molecular organisations.

Structural investigations showed that the enantiomers (**1 a** and **1 b**) formed sheet‐like aggregates, as observed earlier for related dyes C2S4 and C8O4.^8^ Absorbance and fluorescence spectra are similar and in line with the supramolecular structure of the aggregates. Brickwork‐like arrangements of molecules in flat, two‐dimensionally extended sheets can explain this behaviour.^22^ In contrast, spectra of the mesomer (**1 c**) resemble those of special preparations of the reference dyes C8O3 and C8S3, in which bundles of tubes dominate.

Isolated tubes show two longitudinally polarised transitions on the low‐energy side of the absorption spectrum. Thereby, both transitions are radiative. In addition, perpendicularly polarised transitions are observed at higher energy. This results from the double‐layer architecture of the tube walls.5f, 18, 23 In contrast, single‐layered tubes show only one longitudinally polarised, low‐energy transition accompanied by a perpendicularly polarised transition at higher energy and only one emission band in resonance with the low‐energy absorption band.^17^ Eisele et al. explained this interesting effect by the loss of the outer dye monolayer during the bundling process.5e A similar spectral single‐layer signature was observed for preparations of the mesomer (cf. Figures 2 B and 4). Because tube bundles were also detected for **1 c**, we suggest that the same mechanism is valid for this derivative. Volume reconstruction from cryo‐ET data and modelling of highly resolved tube bundle projection images support this concept and provide additional details. The observation of a spectral single‐layer signature for the preparation, with exclusively isolated double‐layered tubes, however, cannot be explained at present.

Amphiphilic dye aggregation in water is driven by a multitude of different forces: amphiphilic and dispersive interactions of alkyl chains, chromophores, and the extended planar π systems, as well as interactions and steric demand of the head groups. Because both the hydrophobic part of the molecules and the chemical composition of the head groups are identical for all new dyes, the differing aggregation behaviour must result from differing interactions of the head groups due to their stereochemistry. Our results reveal that inversion of one stereocentre (enantiomer vs. mesomer) induces the formation of completely different architectures, that is, sheets (Figure 6) and tubes (Figures 8 and 9), respectively, which are associated with particular spectroscopic characteristics.

The chiral enantiomers (**1 a** and **1 b**) form planar, and hence, achiral aggregates, which is a rather unexpected result given the multitude of chiral ultra‐structures reported for chiral compounds in the literature. Thickness measurements indicate that the sheets of the enantiomers are formed by molecular double layers, as expected from the amphiphilic character of the dyes.

**Figure 13:**
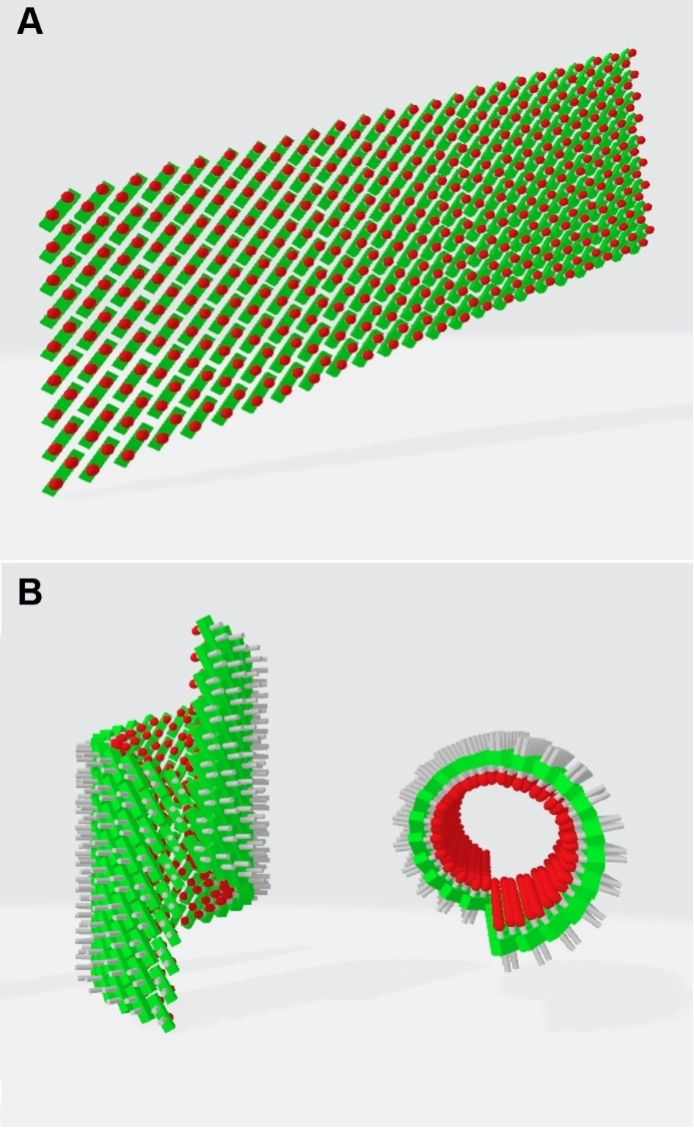
Top: The brickwork arrangement of cyanine dyes constitutes the basic arrangement of molecules in the sheet‐like J aggregates. Bottom: If rolled up into tubes, a helical arrangement of the constituent dyes results, which causes a strong CD signal of the superficially achiral structure.

According to theory, the spectroscopic properties of J aggregates can be explained by a head‐to‐tail arrangement of the transition dipoles of the chromophores. Such an arrangement is realised in a two‐dimensional layer of dye molecules, in which neighbouring molecules are shifted against each other in a brickwork‐like pattern (Figure 13 A).^2^, ^22^ The model explains the occurrence of a single, sharp absorption band and the quasi‐resonant emission. This simple spectroscopic behaviour is observed for many sheet‐like J aggregates, including the related cyanine dyes C2S4 and C8O4, which form extended monolayers and double‐layered sheets, respectively.5b Considering their similar spectroscopic and structural features, we assume a comparable brickwork arrangement for the enantiomers (Figure 13).

The fact that **1 a** and **1 b** form planar structures upon aggregation seems all the more surprising because the related achiral dyes C8O3 and C8S3 form double‐layered tubular structures instead. Some of these tubular structures reveal even helical packing patterns,^6^, ^15^ and the tubes show generally a strong tendency to assemble into twisted rope‐like bundles.5b Hence, such tubes possess a chiral structure and their absorption behaviour can be modelled on this basis.5f, 8c, 9a, 23 The ability of the presented non‐chiral derivatives, that is, the achiral mesomer (**1 c**) and the non‐chiral conformer (**2**), to form tubes suggests a comparable architecture. Considering the Cotton effect of their CD spectra (Figure 4), at least the tubular assemblies of **1 c** are organised in a chiral molecular arrangement (Figure 13 B).

The clear inverse relationship between molecular chirality and helicity of supramolecular architecture asks for a model that can explain the various structures and is consistent with the detected spectra.

The formation of chiral supramolecular structures from achiral or non‐chiral molecules in solution has been reported, but remains rare.^24^ This may derive from the even probabilities to form right‐ or left‐handed assemblies, resulting in a net zero CD signal. Only homochirality can be detected unambiguously by means of CD spectroscopy. Examples include the non‐chiral cyanine dye C8O310a, 11 or the generation of a [1.0] polyglycerol amphiphile reported by Kumar et al.^25^ In the latter case, chiral ultra‐structuring of the self‐assembled tubes was detected by CD only in the case of the *meso* form. The origin of this supramolecular chirality was explained to result from the cooperation of a fixed lateral arrangement of molecules and specific intermolecular hydrogen‐bonding patterns of the hydroxyl groups, which produce a strong helical twist of the molecular stacks only in case of the *meso* form.

**Figure 14:**
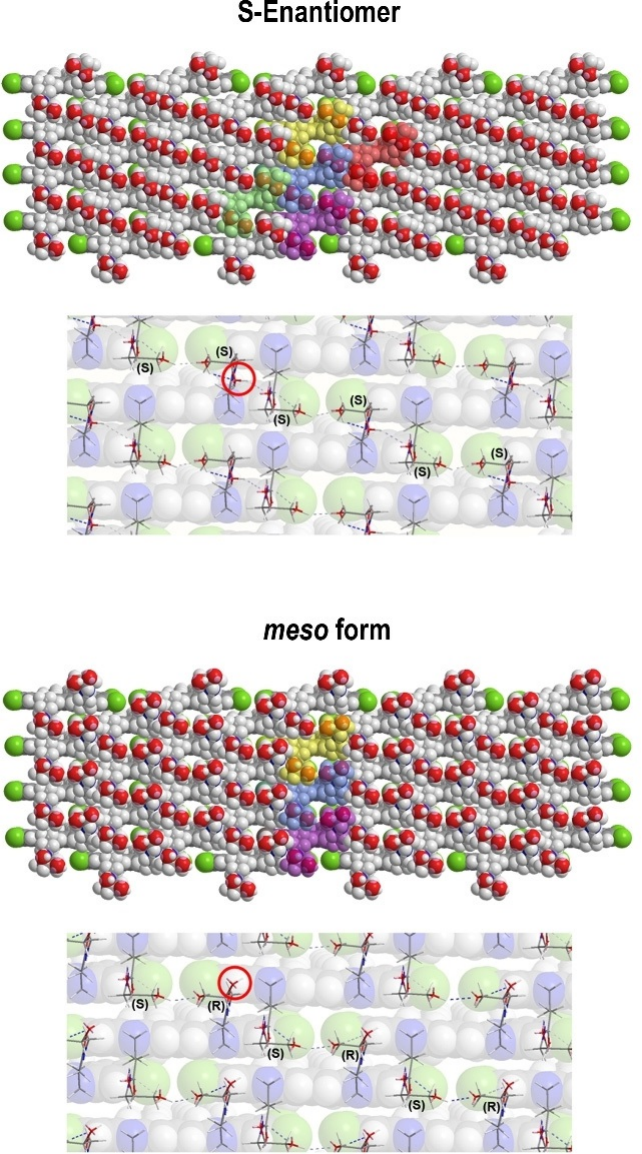
Top: The brickwork arrangement of the *S* enantiomers gives rise to continuous strings of hydroxyl groups (red) running transversely to the chromophore skeletons (coloured in the background). The close up (lower image) shows the OH groups properly situated to form endless hydrogen‐bridge chains. Hence, all dye molecules are connected to four neighbouring molecules (coloured). This stable two‐dimensional arrangement is the unique feature for the chiral enantiomers and cannot be realised by the *meso* form (bottom) due to the different orientation of the respective OH group at the chiral centre (red circles).

We consider a similar reason here. In a brickwork arrangement of the enantiomers forming the planar double layer, the rows of molecules are positioned in a way that neighbouring rows are staggered approximately by half of the chromophore length (Figure 14). The distance between the rows results from the space demand of the chromophore skeletons, which is 0.35–0.4 nm. By placing the enantiomers accordingly, continuous strings of hydroxyl groups can be observed running transversely with respect to the rows of the chromophores (Figure 14, top). A more detailed presentation (below) shows that all OH groups within a row are connected by endless hydrogen‐bond chains. Thus, each dye molecule interacts strongly with four neighbouring molecules to stabilise the planar arrangement. In the case of the mesomer (Figure 14, bottom), however, the inversion of one of the chiral centres prevents linear hydrogen bonds of hydroxyl groups due to their alternating orientation (marked by a red circle). The curved molecular arrangement upon rolling of the sheets into tubes is stabilised by enhanced chromophore interactions.

This comparison gives a good explanation not only of how the introduction of hydrogen‐bonding OH groups can direct the formation of planar sheets in the case of a chiral compound, but also of why the formation of tubular aggregates is preferred by the achiral mesomer. In this respect, as well as regarding the aggregation behaviour of the parent C8O3/S3 dyes, which both only form tubular aggregates, one can suspect that the formation of tubes may be an intrinsic property of the hydrophobic dye skeletons, with their hydrocarbon chains, rather than being provoked by the hydrophilic head groups. The observation that the diameter of monolayered tubes (of the bundles) and of the inner cylinders of the bilayered tubes are identical points at comparable chromophore interactions in both architectures. The initial twist in the monolayered inverted tube bundles, however, gives direct structural evidence for chiral packing in both tubular assembly structures. It is, however, still not clear what drives the assembly process of the mesomer into two structurally different species (individual tubes and bundles).

## Conclusion

We have synthesised a new group of TBC‐based amphiphilic cyanine dye derivatives by converting the anionic carboxylic acid groups into non‐ionic hydroxyl functionalities. The compounds contain aminopropanediol groups, which provide sufficient water solubility and defined chirality. To produce the achiral *meso* form of the dye, a new synthetic route towards asymmetrically functionalised TBC dyes was established.

All compounds formed J aggregates in water. Cryo‐TEM revealed the formation of extended sheet‐like aggregates for the chiral enantiomers **1 a** and **1 b** and individual tubes, with a diameter of 12–13 nm, as well as tube bundles for the mesomer **1 c**. In the case of the conformer **2**, needle‐like crystals predominate by far.

Our findings demonstrate that the supramolecular aggregation behaviour of cyanine dyes can be controlled solely by stereochemistry. The surprising aspect is the inverse relationship between molecular chirality and helicity of the supramolecular architecture. The reason is clearly the special interaction of the non‐ionic hydroxyl functionalities. In the case of the chiral enantiomers **1 a** and **1 b**, extended hydrogen‐bond chains can interconnect molecules in a brickwork arrangement. This yields a two‐dimensional planar network that prevents the spontaneous formation of curved assemblies, such as the tubes and tube bundles formed by **1 c**, which are not capable of forming comparable hydrogen‐bond chains.

Absorption, LD and fluorescence spectra of the tubes indicate a monolayered architecture, although single tubes clearly show a double‐layer wall geometry. In a recent report,5e this apparent contradiction was resolved by the evidence of a dominating population of tube bundles. Supported by cryo‐ET, it was observed that during the hierarchical assembly of the bilayered C8S3 tubes into bundles the outer monolayer was lost. The present cryo‐ET investigation and structural analysis of similar bundled aggregates not only affirms this interpretation, but adds evidence for tight packing of the hydrophobic cylinders.

New insights into the interdependence of stereochemistry and supramolecular aggregation behaviour of cyanine dyes might contribute to the development of specialised, dye‐based materials with predictable properties.

## Experimental Section

### Materials

Dry solvents and chemicals were purchased from Sigma–Aldrich, TCI and abcr Chemicals. Ethyl acetate, hexane and dichloromethane were distilled before use in reactions and in compound purifications. Enantiopure (*R*)‐ and (*S*)‐solketal were purchased from TCI with 98 % *ee* optical purity. C8O3 was obtained from FEW chemicals (Wolfen, Germany). Reactions were monitored by using TLC on silica‐coated aluminium sheets with 60 F254 silica gel or 60 RP‐18 F254S silica gel for reverse‐phase analysis. All intermediates were purified by using normal‐phase column chromatography and automated flash chromatography on a combi Flash *R*_f_ column (Teledyne ISCO) packed with normal silica gel (30 μm). The final products were purified by using reverse‐phase preparative HPLC.

### NMR spectroscopy

^1^H and ^13^C NMR spectra were recorded on a JEOL ECP 500 spectrometer (500 MHz).

### Mass spectrometry

Mass spectra were recorded by using an Agilent 6210 ESI‐TOF spectrometer.

### Sample preparation

Dye stock solutions were prepared from dried solid matter and the respective solvent by vigorously shaking the samples. For spectroscopic measurements, the solutions were diluted, if necessary.

### Spectroscopic measurements

Isotropic absorption spectra (UV/Vis) were measured on a Varian Cary 50 spectrophotometer (Agilent Technologies Inc., Santa Clara, USA); fluorescence spectra were measured on an LS 50B luminescence spectrometer (PerkinElmer, Rodgau, Germany). CD and LD were measured on a J‐810 spectropolarimeter (Jasco Corp., Tokyo, Japan), which could be equipped with a microvolume Couette flow LD cell (Dioptica Scientific Limited, Rugby, Warwickshire, UK) with a 0.5 mm optical path length for the LD measurement.^26^ The LD spectra were independent of the angular velocity of the rotating cell. Rotating velocities up to 3000 rpm, corresponding to shear forces of about 1200 s^−1^, were used. CD measurements were carried out at 20 °C; all other spectroscopic measurements were performed at room temperature ((22±1) °C). Cuvettes for UV/Vis, fluorescence and CD spectroscopy were siliconised before measurements, according to the “Siliconization of Glassware” protocol by using a silicone solution in isopropanol (SERVA Electrophoresis GmbH, Heidelberg, Germany).

### cryo‐TEM

The 200 mesh grids covered with perforated carbon film (R1/4 batch of Quantifoil, MicroTools GmbH, Jena, Germany) were cleaned with chloroform and hydrophilised upon 60 s glow discharging at 8 W in a BAL‐TEC MED 020 device (Leica Microsystems, Wetzlar, Germany). After applying aliquots (5 μL) of the dye solution to the grids, the samples were vitrified by automated blotting and plunge freezing into liquid ethane by using an FEI Vitrobot Mark IV device (Thermo Fisher Scientific Inc., Waltham, MA, USA). The vitrified specimens were transferred under liquid nitrogen to an FEI TALOS L120C electron microscope (Thermo Fisher Scientific Inc., Waltham, MA, USA) by using a Gatan cryo‐holder and stage (model 626, Gatan, Inc., Pleasanton, CA, USA). The microscope was equipped with an LaB6 cathode and operated at 120 kV accelerating voltage. Micrographs were acquired on an FEI Ceta CMOS camera (Thermo Fisher Scientific Inc., Waltham, MA, USA) at a nominal magnification of 36 000×, corresponding to a calibrated pixel size of 4.09 Å per pixel.

### cryo‐ET

Vitrified specimens (see above) intended for cryo‐ET were transferred to the autoloader of an FEI TALOS ARCTICA electron microscope (Thermo Fisher Scientific Inc., Waltham, MA, USA). This microscope is equipped with a high‐brightness field‐emission gun (XFEG) operated at an acceleration voltage of 200 kV. Micrographs were acquired on an FEI Falcon 3 direct electron detector (Thermo Fisher Scientific Inc., Waltham, MA, USA) at a nominal magnification of 28 000×, corresponding to a calibrated pixel size of 3.64 Å per pixel.

Tomography series were recorded in the context of FEI Tomography Software V 4.3.1. The 4096×4096 pixel images were recorded in the tilt angle range of ±65° in 2° increments, with a total electron dose of 180 e Å^−2^. The 3D volume reconstructions were calculated with the help of INSPECT3D Software V4.4 (Thermo Fisher Scientific Inc., Waltham, MA, USA) and visualised with Imod V4.9.10.^27^

### Image processing

Every three consecutive slices of the tomogram along the tube bundle were summed and cropped by using the slicer of Imod V4.9.10.^27^ The cross‐sectional motif of the bundle was boxed off these images by using the boxer module of the EMAN software package.^28^ Alignments, multivariate statistical analysis (MSA) of a total of 232 such motifs, summing, and finally construction of a twisted 3D volume of the bundle with a pitch of 400 nm was performed with the Imagic 5 software package (Image Science Software GmbH, Berlin, Germany). The resulting 3D volume was visualised by using AMIRA Software V2019.1 (Thermo Fisher Scientific Inc., Waltham, MA, USA).

### AFM

AFM was carried out with a Multimode 8 nanoscope with Nanocontroller V (Bruker, Billerica, MA, USA) and equipped with ultra‐sharp PEAKFORCE‐HIRS‐F‐B tips (Bruker), providing a nominal radius of 1 nm and maximum radius of 2 nm. The sample was prepared by deposition of the 0.1 mm aqueous solution of dye (10 μL) on cleaved mica, which was fixed on a circular metal disk with double‐sided tape. The dye assemblies were allowed to settle for about 5 min before the solution was blotted with filter paper until only a thin solution film was left. The sample was then immediately mounted on the AFM scanner and a liquid chamber was assembled and carefully filled with Milli‐Q water to prevent drying of the sample. Imaging was performed with a calibrated cantilever^29^ in PeakForce quantitative nanomechanics (QNM) mode,^30^ to control the loading force on the sample at all times. The maximum loading force used was 500 pN, the resolution was 512 points per line, and the scan rate was 0.7 Hz.

### Synthesis

**Compounds 3 a and 3 b**: Enantiopure solketal (*R* or *S*) was converted into the corresponding phthalimide according to the published procedure from Goubert et al.^31^

**Compound 3 a**: From (*R*)‐(−)‐2,2‐dimethyl‐1,3‐dioxolane‐4‐methanol (4.2 g, 31.8 mmol), the corresponding phthalimide **3 a** was obtained as a colourless solid (7.6 g, 92 %). ^1^H NMR (500 MHz, CDCl_3_): *δ*=7.84 (dd, *J*=5.4, 3.0 Hz, 2 H), 7.71 (dd, *J*=5.5, 3.0 Hz, 2 H), 4.49–4.38 (m, 1 H), 4.06 (dd, *J*=8.7, 6.2 Hz, 1 H), 3.92 (dd, *J*=13.8, 6.9 Hz, 1 H), 3.84 (dd, *J*=8.7, 5.1 Hz, 1 H), 3.71 (dd, *J*=13.8, 5.3 Hz, 1 H), 1.43 (s, 3 H), 1.30 ppm (s, 3 H); ^13^C NMR (126 MHz, CDCl_3_): *δ*=168.3, 134.2, 132.1, 123.5, 110.0, 73.4, 67.5, 41.1, 27.0, 25.5 ppm; HRMS: *m*/*z* calcd for C_14_H_15_NNaO_4_^+^: 284.0893; found: 284.0898.

**Compound 3 b**: From (*S*)‐(+)‐2,2‐dimethyl‐1,3‐dioxolane‐4‐methanol (2.0 g, 15.1 mmol), the corresponding phthalimide **3 b** was obtained as a colourless solid (3.3 g, 83 %). 1H NMR (500 MHz, CDCl_3_): *δ*=7.84 (dd, *J*=5.5, 3.0 Hz, 2 H), 7.71 (dd, *J*=5.5, 3.0 Hz, 2 H), 4.49–4.38 (m, 1 H), 4.06 (dd, *J*=8.7, 6.2 Hz, 1 H), 3.92 (dd, *J*=13.8, 6.9 Hz, 1 H), 3.84 (dd, *J*=8.7, 5.1 Hz, 1 H), 3.71 (dd, *J*=13.8, 5.3 Hz, 1 H), 1.43 (s, 3 H), 1.30 ppm (s, 3 H); ^13^C NMR (126 MHz, CDCl_3_): *δ*=168.3, 134.2, 132.1, 123.5, 110.0, 73.4, 67.5, 41.1, 27.0, 25.5 ppm; HRMS *m*/*z* calcd for C_14_H_15_NNaO_4_^+^: 284.0893; found: 284.0891.

**Compounds 4 a and 4 b**: 2‐[(2,2‐Dimethyl‐1,3‐dioxolan‐4‐yl)methyl]isoindoline‐1,3‐dione **3** (**a** or **b**) were reacted according to a procedure published in the literature.^31^

**Compound 4 a**: From (*S*)‐phthalimide **3 a** (1.4 g, 5.4 mmol), the corresponding amine **4 a** was obtained as a pale‐yellow liquid (0.3 g, 43 %). ^1^H NMR (500 MHz, CD_3_OD): *δ*=4.14 (qd, *J*=6.4, 4.8 Hz, 1 H), 4.05 (dd, *J*=8.3, 6.4 Hz, 1 H), 3.65 (dd, *J*=8.3, 6.3 Hz, 1 H), 2.81–2.64 (m, 2 H), 1.39 (s, 3 H), 1.33 ppm (s, 3 H); ^13^C NMR (126 MHz, CD_3_OD): *δ*=110.3, 78.1, 68.0, 45.2, 27.2, 25.6 ppm; HRMS: *m*/*z* calcd for C_6_H_14_NO_2_^+^: 132.1019; found: 132.1008.

**Compound 4 b**: From (*R*)‐phthalimide **3 b** (1.0 g, 3.9 mmol), the corresponding amine **4 b** was obtained as a pale‐yellow liquid (0.28 g, 55 %). ^1^H NMR (500 MHz, CD_3_OD): *δ*=4.13 (qd, *J*=6.4, 4.8 Hz, 1 H), 4.04 (dd, *J*=8.2, 6.4 Hz, 1 H), 3.65 (dd, *J*=8.2, 6.4 Hz, 1 H), 2.80–2.64 (m, 2 H), 1.39 (s, 3 H), 1.33 ppm (s, 3 H); ^13^C NMR (126 MHz, CD_3_OD): *δ*=110.3, 78.2, 68.1, 45.3, 27.2, 25.6 ppm; HRMS: *m*/*z* calcd for C_6_H_14_NO_2_^+^: 132.1019; found: 132.1037.

**Compounds 5 a and 5 b**: DIPEA (0.518 mmol) was added to a solution of C8O3 (30 mg, 0.037 mmol), HATU (48 mg, 0.126 mmol) and solketal amine **4** (**a** or **b**; 48.5 mg, 0.370 mmol) in DMF (3 mL) and the mixture was stirred at RT for 2 h. After removal of the solvent in vacuum, the residue was dissolved in CH_2_Cl_2_ and washed with water three times. The combined organic phases were concentrated, and the residue was purified by automated column chromatography (CH_2_Cl_2_/methanol, 0–5 %).

**Compound 5 a**: Solketal amine **4 a** was reacted according to the above‐described procedure and dye **5 a** was obtained as a red solid (36 mg, 91 %). ^1^H NMR (700 MHz, CD_3_OD): *δ*=8.00 (s, 1 H), 7.76 (s, 2 H), 7.73 (s, 2 H), 4.31 (dt, *J*=14.9, 7.5 Hz, 8 H), 4.10 (p, *J*=5.8 Hz, 2 H), 3.97 (dd, *J*=8.4, 6.3 Hz, 2 H), 3.60 (dd, *J*=8.4, 5.9 Hz, 2 H), 3.28–3.20 (m, 4 H), 2.39 (t, *J*=6.7 Hz, 4 H), 2.14 (p, *J*=6.9 Hz, 4 H), 1.87 (p, *J*=7.4 Hz, 4 H), 1.45–1.21 (m, 32 H), 0.86 ppm (t, *J*=7.0 Hz, 6 H); ^13^C NMR (176 MHz, CD_3_OD): *δ*=174.4, 151.4, 144.1, 133.8, 133.6, 128.7, 116.6, 112.6, 110.5, 75.9, 68.2, 46.3, 45.7, 43.0, 32.9, 32.7, 30.8, 30.4, 29.1, 27.7, 27.2, 25.6, 24.6, 23.7, 14.4 ppm; HRMS: *m*/*z* calcd for C_53_H_77_Cl_4_N_6_O_6_^+^: 1035.4624; found: 1035.4713.

**Compound 5 b**: Solketal amine **4 b** (48.5 mg, 0.370 mmol) was reacted according to the above‐described procedure and dye **5 b** was obtained as a red solid (32 mg, 81 %). ^1^H NMR (700 MHz, CD_3_OD): *δ*=8.01 (s, 1 H), 7.76 (s, 2 H), 7.73 (s, 2 H), 5.82 (d, *J*=13.3 Hz, 2 H), 4.31 (dt, *J*=14.0, 8.0 Hz, 8 H), 4.10 (p, *J*=5.8 Hz, 2 H), 3.97 (dd, *J*=8.4, 6.3 Hz, 2 H), 3.60 (dd, *J*=8.4, 5.9 Hz, 2 H), 3.26–3.21 (m, 4 H), 2.38 (t, *J*=6.8 Hz, 4 H), 2.14 (p, *J*=7.0 Hz, 4 H), 1.87 (p, *J*=7.3 Hz, 4 H), 1.45–1.21 (m, 32 H), 0.85 ppm (t, *J*=6.9 Hz, 6 H); ^13^C NMR (176 MHz, CD_3_OD): *δ*=174.5, 151.3, 133.8, 133.6, 128.9, 128.6, 112.5, 110.5, 75.9, 68.2, 46.3, 45.7, 43.0, 32.9, 32.7, 30.4, 30.3, 29.1, 27.6, 27.2, 25.6, 24.6, 23.7, 14.4 ppm; HRMS: *m*/*z* calcd for C_53_H_77_Cl_4_N_6_O_6_^+^: 1035.4624; found: 1035.4797.

**Compounds 1 a and 1 b**: Hydrogen chloride (1.301 mmol) was added to a solution of dye **5** (**a** or **b**; 0.033 mmol) in methanol (100 mL) and the mixture was stirred at RT for 3 h. The solvents were removed under vacuum by distillation with toluene and the crude product was lyophilised. The obtained residue was purified by means of reversed‐phase column chromatography with water/acetonitrile (75 %) and 50 mm ammonium formate.

**Compound 1 a**: Dye **5 a** (35 mg, 0.033 mmol) was reacted according to the procedure described above. Dye **1 a** was obtained as a red solid (31.4 mg, 96 %). ^1^H NMR (700 MHz, CD_3_OD): *δ*=8.46 (m, 1 H), 8.40–8.38 (m, 2 H), 8.36 (m, 1 H), 7.25 (s, 1 H), 4.71–4.53 (m, 8 H), 3.65–3.58 (m, 3 H), 3.49–3.43 (m, 3 H), 3.20 (m, 2 H), 3.01–2.99 (m, 1 H) 2.88–2.86 (m, 1 H), 2.52–2.41 (m, 4 H), 2.29–2.15 (m, 4 H), 2.04–1.91 (m, 4 H), 1.42–1.30 (m, 20 H), 0.92–0.88 ppm (m, 6 H); ^13^C NMR (176 MHz, MeOD): *δ*=173.2, 150.5, 148.0, 141.4, 136.5, 131.8, 131.6, 131.4, 131.2, 131.1, 130.7, 115.4, 115.1, 70.5, 63.6, 55.8, 42.0, 31.5, 29.2, 28.8, 26.3, 22.4, 13.1 ppm; HRMS: *m*/*z* calcd for C_47_H_69_Cl_4_N_6_O_6_^+^: 955.3998; found: 955.4044.

**Compound 1 b**: Dye **5 b** (30 mg, 0.028 mmol) was reacted according to the procedure described above. Dye **1 b** was obtained as a red solid (26.1 mg, 94 %). ^1^H NMR (700 MHz, CD_3_OD): *δ*=8.44 (s, 1 H), 8.38 (s, 1 H), 8.37 (s, 1 H), 8.33 (s, 1 H), 7.23 (s, 1 H), 4.68–4.49 (m, 8 H), 3.62–3.56 (m, 2 H), 3.44–3.42 (m, 4 H), 3.26–3.25 (m, 1 H) 3.17–3.14 (m, 1 H), 3.00–2.97 (m, 1 H), 2.86–2.83 (m, 1 H), 2.52–2.42 (m, 4 H), 2.25–2.13 (m, 4 H), 1.99–1.90 (m, 4 H), 1.52–1.29 (m, 20, H), 0.90–0.88 ppm (m, 6 H); ^13^C NMR (176 MHz, CD_3_OD): *δ*=174.6, 174.1, 151.9, 149.4, 143.0, 132.9, 132.9, 132.8, 132.7, 132.4, 132.4, 132.4, 132.3, 116.4, 116.3, 116.2, 71.9, 71.8, 65.0, 47.9, 47.3, 47.0, 43.4, 43.1, 33.0, 32.4, 32.1, 30.5, 30.4, 30.4, 30.3, 27.7, 25.9, 25.4, 23.7, 14.4 ppm; HRMS: *m*/*z* calcd for C_47_H_69_Cl_4_N_6_O_6_^+^: 955.3998; found: 955.4044.

**Compound 2**: DIPEA (88.2 μL, 0.518 mmol) was added to a solution of C8O3 (30 mg, 0.037 mmol), HATU (48 mg, 0.126 mmol) and serinol (34 mg, 0.370 mmol) in DMF (3 mL) and the mixture was stirred at RT for 2 h. After removal of the solvent under vacuum, the residue was purified by means of reversed‐phase column chromatography with water/acetonitrile (75 %) and 50 mm ammonium formate to give compound **2** as a red solid (16 mg, 44 %). ^1^H NMR (700 MHz, CD_3_OD): *δ*=8.50 (s, 1 H), 7.99 (s, 1 H), 7.78 (s, 2 H), 7.74 (s, 2 H), 4.35–4.28 (m, 12 H), 3.92 (p, *J*=5.6 Hz, 2 H), 3.60 (dd, *J*=11.0, 5.4 Hz, 4 H), 3.57 (dd, *J*=11.1, 5.8 Hz, 4 H), 2.42 (t, *J*=6.9 Hz, 4 H), 2.14 (p, *J*=7.2 Hz, 4 H), 1.87 (p, *J*=6.9 Hz, 4 H), 1.44–1.21 ppm (m, 20 H), 0.86 (t, *J*=6.9 Hz, 6 H); ^13^C NMR (176 MHz, CD_3_OD): *δ*=174.4, 170.3, 151.33, 151.27, 133.8, 133.6, 128.7, 112.6, 62.1, 54.5, 46.3, 45.7, 33.0, 32.9, 30.4, 30.3, 29.1, 27.7, 24.8, 23.7, 14.4 ppm; HRMS *m*/*z* calcd for C_47_H_69_C_l4_N_6_O_6_^+^: 955.3998; found: 955.4007.

**Compound 6**: The required amount of NaOH (1.09 g, 27.3 mmol, 1.1 equiv) was added to a stirred solution of 5,6‐dichlorobenzimidazole (5 g, 24.8 mmol, 1 equiv) in DMSO. The reaction solution was stirred for 2 h at room temperature. Thereafter, the calculated amount of ethyl 4‐bromobutanoate (5.82 g, 28.5 mmol, 1.2 equiv) was added and the reaction mixture was left to stir for 48 h at room temperature. Progress of the reaction was monitored by TLC with methanol/dichloromethane as the eluent. On completion of the reaction, the mixture was suspended in water and ethyl acetate (3×30 mL). The combined organic layers were dried over anhydrous sodium sulfate and the solvent was evaporated to yield the crude product, which was purified through column chromatography with CH_2_Cl_2_ and methanol to give compound **6** as a white solid (95 %). ^1^H NMR (500 MHz, [D_3_]methanol): *δ*=7.66 (s, 1 H), 7.61 (s, 1 H), 4.20 (t, *J=7.45*, 3 H), 4.07–4.02 (q, 2 H), 2.59 (s, 3 H), 2.41 (t, *J=6.70*, 2 H), 2.08–2.02 (m, 2 H), 1.20 ppm (t, *J=7.15*, 3 H); ^13^C NMR (126 MHz, [D_3_]methanol): *δ*=174.2, 156.0, 142.3, 135.6, 127.1, 126.8, 120.1, 112.7, 61.7, 44.2, 31.5, 25.4, 14.4, 13.5 ppm; HRMS: *m*/*z* calcd for C_14_H_17_Cl_2_N_2_O_2_^+^: 315.0361; found: 315.0589.

**Compound 7**: Compound **6** (2 g, 6.34 mmol, 1 equiv) was liquefied at 150 °C. 1‐Bromodecane (7.0 g, 31.7 mmol, 5 equiv) was added to the reaction flask and the reaction mixture was left stirring at 150 °C for 6 h. After completion of the reaction (as indicated by TLC with methanol/dichloromethane as the eluent), the reaction mixture was extracted with water and dichloromethane (3×50 mL). The combined organic layers were dried over anhydrous sodium sulfate and the solvent was evaporated to yield the crude product, which was further purified through column chromatography with CH_2_Cl_2_ and methanol as the eluent to give compound **7** as a light‐yellowish solid (72 %). ^1^H NMR (500 MHz, CDCl_3_): *δ*=8.22 (s, 1 H), 7.81 (s, 1 H), 4.66 (t, *J=7.75*, 3 H), 4.44 (t, *J*=7.45, 3 H), 3.99–3.95 (q, 2 H), 2.55 (t, *J=6.50*, 2 H), 2.13–2.07 (m, 2 H), 1.82–1.76 (m, 2 H), 1.36–1.30 (m, 2 H), 1.25–1.11 (m, 13 H), 0.77 ppm (t, *J=4.55*, 3 H); ^13^C NMR (126 MHz, CDCl_3_): *δ*=172.7, 153.1, 131.4, 131.3, 130.3, 130.1, 114.9, 114.0, 77.4, 77.2, 76.9, 60.7, 47.1, 45.9, 31.6, 30.2, 29.0, 28.9, 26.6, 23.9, 22.4, 14.0, 13.9, 13.0 ppm; HRMS: *m*/*z* calcd for C_22_H_34_Cl_2_N_2_O_2_^+^: 428.1914; found: 428.1876.

**Compound 8**: Compound **7** (1 g 1.8 mmol, 1 equiv) was stirred with a 1:1 mixture of HBr (48 %) and water at 120 °C for 15 h. Progress of the reaction was monitored by TLC with methanol/dichloromethane as the eluent. On completion of the reaction, the reaction mixture was cooled to room temperature. The precipitate was filtered and washed with aqueous hydrobromic acid (5 % w/w) to yield compound **8** as a light‐yellowish solid (98 %). ^1^H NMR (500 MHz, [D_3_]methanol): *δ*=8.35 (s, 1 H), 8.30 (s, 1 H), 4.55 (t, *J*=7.77 Hz, 2 H), 4.48 (t, *J*=7.65 Hz, 2 H), 2.98 (s, 3 H), 2.54 (t, *J*=6.45 Hz, 2 H), 2.19–2.13 (m, 2 H), 1.93–1.86 (m, 2 H), 1.48–1.32 (m, 12 H), 0.91 ppm (t, *J*=6.85 Hz, 3 H); ^13^C NMR (126 MHz, [D_3_]methanol): *δ*=174.7, 153.2, 130.7, 114.6, 46.0, 45.0, 31.6, 29.6, 28.9, 28.7, 26.2, 23.5, 22.4, 13.0, 9.8 ppm; HRMS: *m*/*z* calcd for C_20_H_30_Cl_2_N_2_O_2_^+^: 400.1608; found: 400.1601.

**Compound 9**: The required amount of KOH (0.88 g, 15.8 mmol, 2 equiv) was added to a stirred solution of compound **6** (2.5 g, 7.9 mmol, 1 equiv) in ethanol. The reaction solution was stirred for 12 h at refluxing temperature. Progress of the reaction was indicated by TLC with methanol/dichloromethane as the eluent. On completion, the reaction mixture was neutralised by using Dowex‐50 cation‐exchange resin. The resin was filtered and the filtrate was concentrated under reduced pressure to give compound **9** as an off‐white solid (95 %). ^1^H NMR (500 MHz, [D_3_]methanol): *δ*=7.76 (s, 1 H), 7.66 (s, 1 H), 4.24 (t, *J*=7.55 Hz, 2 H), 2.60 (s, 3 H), 2.08, 2.06–2.02 ppm (m, 2 H); ^13^C NMR (126 MHz, [D_3_]methanol): *δ*=174.8, 154.8, 140.9, 134.4, 125.8, 118.8, 111.5, 42.9, 29.9, 24.3, 12.1 ppm; HRMS: *m*/*z* calcd for C_12_H_13_Cl_2_N_2_O_2_: 287.0266; found: 287.0276.

**Compound 10**: (*S*)‐Solketal amine (**4 b**), EDC**⋅**HCl (2.0 g, 10.4 mmol, 1.5 equiv) and DMAP (0.45 g, 3.4 mmol, 0.5 equiv) were added to a stirred solution of compound **9** (2.0 g, 6.94 mmol, 1 equiv) in DMF (30 mL) at room temperature. The reaction mixture was left to stir for 24 h. Progress of the reaction was monitored by TLC with methanol/dichloromethane as the eluent. On completion, the mixture was suspended in water and CH_2_Cl_2_ (3×30 mL). The combined organic layers were dried over anhydrous sodium sulfate and the solvent was evaporated to yield the crude product, which was purified through column chromatography with CH_2_Cl_2_ and methanol as the eluent to give compound **10** as a white solid (75 %). ^1^H NMR (500 MHz, CDCl_3_): *δ*=7.64, 7.37, 7.26, 6.11 (t, *J*=5.6 Hz, 1 H), 4.19–4.14 (m, 1 H), 4.10 (t, *J*=7.2 Hz, 2 H) 4.02–3.99 (m, 1 H), 3.59–3.56 (m, 1 H), 3.55–3.51 (m, 1 H), 3.22–3.17 (m, 1 H), 2.52 (s, 3 H), 2.18 (t, *J*=6.8, 3 H), 2.08–2.03 (m, 2 H), 1.35 (s, 3 H), 1.28 ppm (s, 3 H); ^13^C NMR (126 MHz, CDCl_3_): *δ*=171.4, 153.8, 142.0, 134.5, 126.0, 125.8, 120.1, 110.7, 109.5, 77.4, 77.2, 76.9, 74.5, 66.8, 43.2, 42.0, 32.0, 26.9, 25.1, 24.9, 13.9 ppm; MS: *m*/*z* calcd for C_18_H_24_Cl_2_N_3_O_3_^+^: 400.1218; found: 400.1116.

**Compound 11**: Compound **10** (1.5 g, 6.34 mmol, 1 equiv) was liquefied at 150 °C. 1‐Bromodecane (7.0 g, 31.7 mmol, 5 equiv) was added to the reaction flask and the reaction mixture was left to stir at 150 °C for 6 h. Progress of the reaction was monitored by TLC with methanol/dichloromethane as the eluent. Upon completion of the reaction, the mixture was extracted with water and dichloromethane (3×50 mL). The combined organic layers were dried over anhydrous sodium sulfate and the solvent was evaporated to yield the crude product, which was further purified through column chromatography with CH_2_Cl_2_ and methanol as the eluent to give compound **11** as a white solid (65 %). ^1^H NMR (500 MHz, CDCl_3_): *δ*=8.18 (s, 1 H), 7.77 (s, 1 H), 7.50 (t, *J*=5.9 Hz, 1 H), 4.62 (t, *J*=7.1 Hz, 2 H), 4.42 (t, *J*=7.5 Hz, 2 H), 4.16–4.12 (m, 1 H), 3.98–3.95 (m, 1 H), 3.66–3.64 (m, 1 H), 3.26 (t, *J*=5.9 Hz, 2 H), 2.53 (t, *J*=6.9 Hz, 2 H), 2.23–2.18 (m, 2 H), 1.87–1.81 (m, 2 H), 1.37–1.22 (m, 18 H), 0.83 ppm (t, *J*=6.9 Hz, 3 H); ^13^C NMR (126 MHz, CDCl_3_): *δ*=172.2, 153.0, 131.8, 130.4, 130.3, 115.1, 114.0, 109.3, 77.4, 77.2, 76.9, 74.4, 67.6, 47.2, 46.3, 42.0, 32.1, 31.7, 29.09, 29.05, 27.0, 26.8, 25.4, 24.8, 22.6, 14.1, 12.9 ppm; HRMS: *m*/*z* calcd for C_26_H_40_C_l2_N_2_O_3_^+^: 512.2455; found: 512.2441.

**Compound 12**: Compounds **8** (0.5 g, 0.001 mmol, 1 equiv) and **11** (0.5 g, 0.008 mmol, 1 equiv) were weighed into a round‐bottomed flask. CHI_3_ (0.18 g, 0.0004 mmol, 0.45 equiv) and DBU (1.1 g, 0.007, 7 equiv) were added to the reaction flask followed by the addition of methanol (25 mL) as a solvent. The reaction mixture was left to stir at room temperature for 48 h. Progress of the reaction was monitored by TLC with methanol/dichloromethane as the eluent. Upon completion of the reaction, the mixture was the crude product, which was purified through column chromatography with CH_2_Cl_2_ and methanol to give compound **12** as a white solid (11 %). ^1^H NMR (500 MHz, [D_4_]MeOH): *δ*=8.34 (s, 2 H), 8.29 (s, 2 H), 4.55–4.47 (m, 8 H), 4.12–4.07 (m, 1 H), 4.01–3.98 (m, 1 H), 3.62–3.60 (m, 1 H), 3.24–3.22 (d, 2 H), 2.45 (t, *J*=6.7 Hz, 4 H), 2.21–2.16 (m, 4 H), 1.93–1.87 (m, 4 H), 1.50–1.45 (m, 4 H), 1.43–1.30 (m, 20 H), 0.90 ppm (t, *J*=6.8 Hz, 6 H); ^13^C NMR (126 MHz, [D_3_]methanol): *δ*=173.0, 130.79, 130.76, 130.72, 114.7, 114.6, 109.2, 74.5, 66.9, 48.2, 48.1, 48.0, 47.94, 47.93, 47.92, 47.91, 47.88, 47.85, 47.84, 47.83, 47.7, 47.5, 47.4, 47.2, 46.2, 45.4, 41.7, 31.6, 31.5, 29.0, 28.9, 28.8, 26.3, 25.9, 24.3, 24.0, 22.4, 13.1 ppm; HRMS: *m*/*z* calcd for C_53_H_77_Cl_4_N_6_O_6_^+^: 922.3783; found: 922.3812.

**Compound 13**: (*R*)‐Solketal amine (0.114 g, 0.086 mmol, 1.5 equiv), EDC**⋅**HCl (0.172 g, 0.086 mmol, 1.5 equiv) and DMAP (0.035 g, 0.028 mmol, 0.5 equiv) were added to a stirred solution of **12** (0.060 g, 0.005 mmol, 1 equiv) in DMF (30 mL) at room temperature. The reaction mixture was left to stir for 24 h. Progress of the reaction was monitored by TLC with methanol/dichloromethane as the eluent. Upon completion of the reaction, the mixture was suspended in water and CH_2_Cl_2_ (3×30 mL). The combined organic layers were dried over anhydrous sodium sulfate and the solvent was evaporated to yield the crude product, which was purified through column chromatography with CH_2_Cl_2_ and methanol as the eluent to give **13** as a red solid (77 %). ^1^H NMR (500 MHz, [D_4_]MeOH): *δ*=8.01 (s, 1 H), 7.78 (s, 2 H), 7.75 (s, 2 H), 4.36–4.30 (m, 8 H), 4.13–4.11 (m, 2 H), 4.00–3.97 (dd, *J*=8.4, 6.3 Hz, 2 H), 3.64–3.61 (dd, J=*J*=8.4, 6.0 Hz, 2 H), 3.27–3.25 (m, 2 H), 2.41 (t, *J*=6.7 Hz, 4 H), 2.19–2.13 (p, *J*=6.7 Hz, 4 H), 1.92–1.86 (p, *J*=6.4, 4 H), 1.42–1.27 (m, 32 H), 0.87 ppm (t, *J*=6.8 Hz, 6 H); ^13^C NMR (126 MHz, CDCl_3_): *δ*=174.5, 133.8, 133.5, 128.6, 112.5, 110.5, 75.8, 68.2, 49.5, 49.2, 49.0, 48.8, 48.5, 46.4, 45.7, 43.0, 32.9, 32.8, 30.4, 30.3, 29.1, 27.6, 27.2, 25.6, 24.7, 23.7, 14.4 ppm; HRMS: *m*/*z* calcd for C_53_H_77_Cl_4_N_6_O_6_^+^: 1035.4624; found: 1035.4696.

**Compound 1 c**: HCl (1.301 mmol) was added to a solution of **13** (0.033 mmol) in methanol (100 mL) and the mixture was stirred at RT for 3 h. The solvents were removed under vacuum by distillation with toluene, and the crude product was lyophilised and further purified by means of reverse‐phase HPLC with 95 % MeCN and water as the eluent and ammonium formate as a modifier. The desired compound **1 c** was obtained as a red solid (37 %). ^1^H NMR (600 MHz, [D_4_]MeOH): *δ*=8.50 (s, 1 H), 7.96 (s, 1 H), 7.74 (s, 2 H), 7.71 (s, 2 H), 4.30–4.27 (m, 8 H), 3.99–3.98 (m, 1 H), 3.65–3.61 (m, 3 H), 3.43–3.42 (m, 4 H), 3.15–3.12 (m, 2 H), 2.38 (m, 4 H), 2.14 2.09 (m 4 H), 1.87–1.82, (m, 4 H), 1.36–1.24 (m, 20 H), 0.83 ppm (t, *J*=6.8 Hz, 6 H); ^13^C NMR (176 MHz, MeOD): *δ*=173.3, 149.9, 147.9, 136.4, 132.8, 132.5, 132.3, 131.5, 131.1, 130.9, 127.4, 122.3, 115.2, 111.2, 70.5, 63.6, 45.0, 44.1, 42.0, 31.5, 29.3, 29.1, 29.0, 28.9, 28.7, 27.6, 26.4, 26.1, 25.1, 24.7, 23.4, 22.3, 13.0 ppm; HRMS: *m*/*z* calcd for C_47_H_69_Cl_4_N_6_O_6_^+^: 955.3998; found: 955.3981.

## Conflict of interest

The authors declare no conflict of interest.

## Supporting information

As a service to our authors and readers, this journal provides supporting information supplied by the authors. Such materials are peer reviewed and may be re‐organized for online delivery, but are not copy‐edited or typeset. Technical support issues arising from supporting information (other than missing files) should be addressed to the authors.

Supplementary

Supplementary
