# Supplementary material for: Stereochemistry‐Controlled Supramolecular Architectures of New Tetrahydroxy‐Functionalised Amphiphilic Carbocyanine Dyes
Source: Chemistry. 2020 Apr 30;26(30):6919–34. doi: 10.1002/chem.201905745 (PMC7317399; doi:10.1002/chem.201905745)
Supplement: Supplementary file 1 — Supplementary [file CHEM-26-6919-s001.pdf]

Chemistry–A European Journal

Supporting Information

Stereochemistry-Controlled Supramolecular Architectures of New Tetrahydroxy-Functionalised Amphiphilic Carbocyanine Dyes

Boris Schade+,[a] Abhishek Kumar Singh+,*[b] Virginia Wycisk,[b] Jose Luis Cuellar-Camacho,[b] Hans von Berlepsch,[a] Rainer Haag,[b] and Christoph Böttcher*[a]

Stereochemistry controlled supramolecular architectures of novel tetrahydroxy function-alized amphiphilic carbocyanine dyes

Boris Schade†,[1] Abhishek Kumar Singh†*,[2] Virginia Wycisk,[2] Jose Luis Cuellar-Camacho,[2] Hans v. Berlepsch,[1] Rainer Haag,[2] and Christoph Böttcher*[1]

Abbreviations of dyes

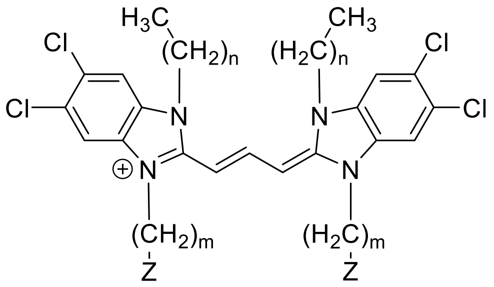

	Z	n	m
TDBC (C2S4)	$-SO_3$	1	4
C8S3	$-SO_3$	7	3
C8O3	-COOH	7	3

Synthesis

Synthetic route

Synthesis of targeted amphiphlic cyanine dye derivatives (compounds **1a, 1b and 1c**) has been accomplished by following two different synthetic approaches. A straightforward route has been employed to obtain the enantiomers. Stereo chemistry of the head groups has been maintained from the very first step in which R and S solketol has been converted to their respective solketal amine, *i.e*. **4a** and **4b** by following the synthetic route depicted in Scheme SI 1. Coupling of the enantiomers of solketal amine to commercially available 5,5',6,6'-tetrachloro-1,1'dialkyl-3,3'-bis(3-carboxypropyl)-benzimidacarbocycanine (C8O3) was followed by deprotection of the acetal head groups under mild acidic condition to give the enantiomers **1a** and **1b** (Scheme SI 2).

[1] Forschungszentrum für Elektronenmikroskopie und Gerätezentrum BioSupraMol, Institut für Chemie und Biochemie, Freie Universität Berlin, Fabeckstraße 36a, 14195 Berlin, Germany

[2] Institut für Chemie und Biochemie, Organische Chemie, Freie Universität Berlin, Takustrasse 3, 14195 Berlin, Germany

† These authors contributed equally to this work

Another synthetic route had to be employed for the meso form (**1c**) which is bearing two oppositely configured head groups. Here, two differently functionalized benzimidazoles had to be coupled. For the first one, commercially available benzimidazole was first treated with ethyl 4-bromobutanoate yielding monosubstituted **6** which was then *N*-alkylated with 1-bromo octane to obtain the *N,N*-disubstituted benzimidazole **7**. Finally, **7** was hydrolysed in presence of HBr yielding disubstituted benzimidazole **8**. For the second one, hydrolysis of **6** yielding acid **9** was followed by amidation of the acid group with (*S*)-solketal amine (**4a**) yielding **10** which *N*-alkylation with 1-bromo octane (Scheme SI 2) gave **11**. Both benzimidazoles **8** and **11** were then coupled in the presence of DBU and CH_3I in methanolic solution resulting in asymmetrically substituted dye **12**. As expected, three different products having differing polarities were formed as can be seen from TLC (Scheme SI 3). According to the polarity order, desired monoacid dye derivative **12** was isolated by column chromatography. After purification, **12** was coupled with (*R*)-solketal amine (**4b**) followed by hydrolysis of the acetal group under acid conditions, which finally yielded the meso-cyanine dye (**1c**) (Scheme SI 2).

Conformer (**2**) was readily synthesized just by direct N-acylation using HATU in the presence of DIPEA as coupling reagent.

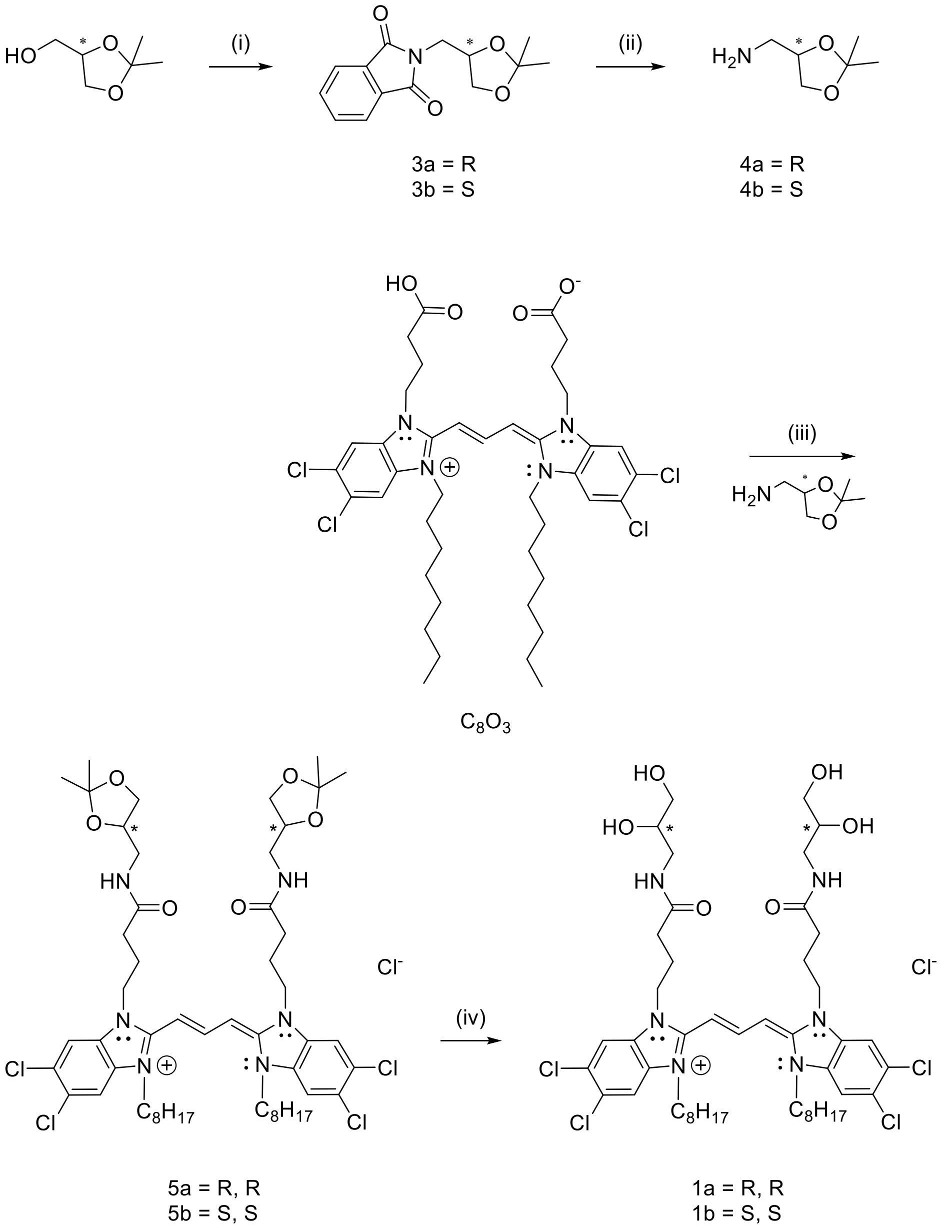

Scheme SI 1: Synthesis of the enantiomers (1a and 1b); i) PPh_3, phthalimide, DEAD, THF, r.t., 20 h; ii) NH_2-NH_2, H_2O, MeOH, reflux, 4-5 h; iii) HATU, DIPEA, DMF, r.t., 2 h; iv) HCl, MeOH, r.t., 2-5 h.

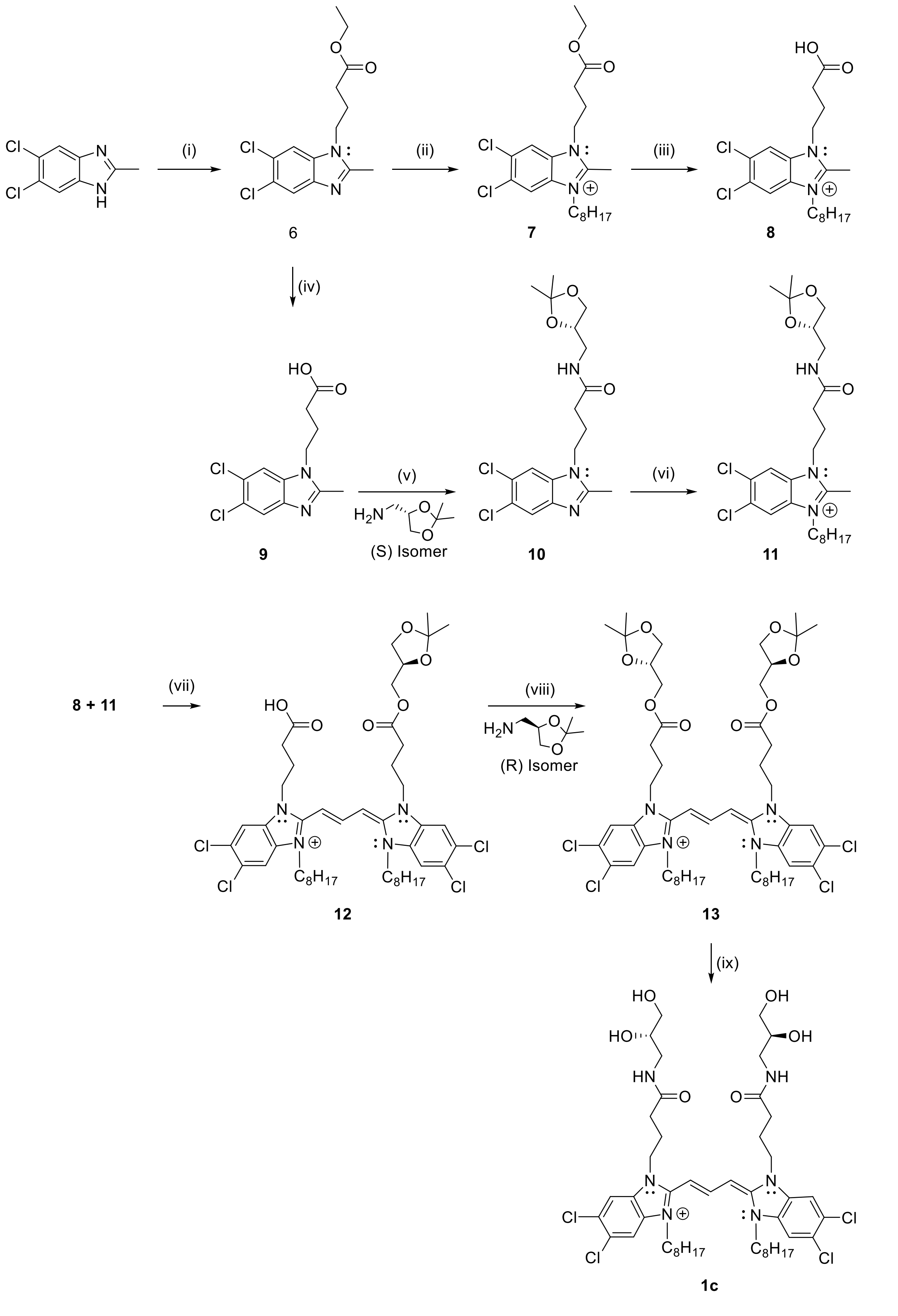

Scheme SI 2: Top) Synthetic route to achiral mesomer (1c); i) Ethyl-bromo acetatae, NaOH, DMSO, r.t., 48 h; ii) 1-bromodecane, 150 °C, 6h; iii) HBr 48%, water, 120 °C, iv) KOH, ethanol, reflux, 12 h; v) EDC.HCl, DMAP,

DMF, 24 h, rt; vi) 1-bromodecane, 150 °C, vii) DBU, CHI_3, methanol, r.t., 48 h; viii) (R) solketal, EDC.HCl, DMAP, DMF, 24 h, r.t., ix) HCl, methanol.

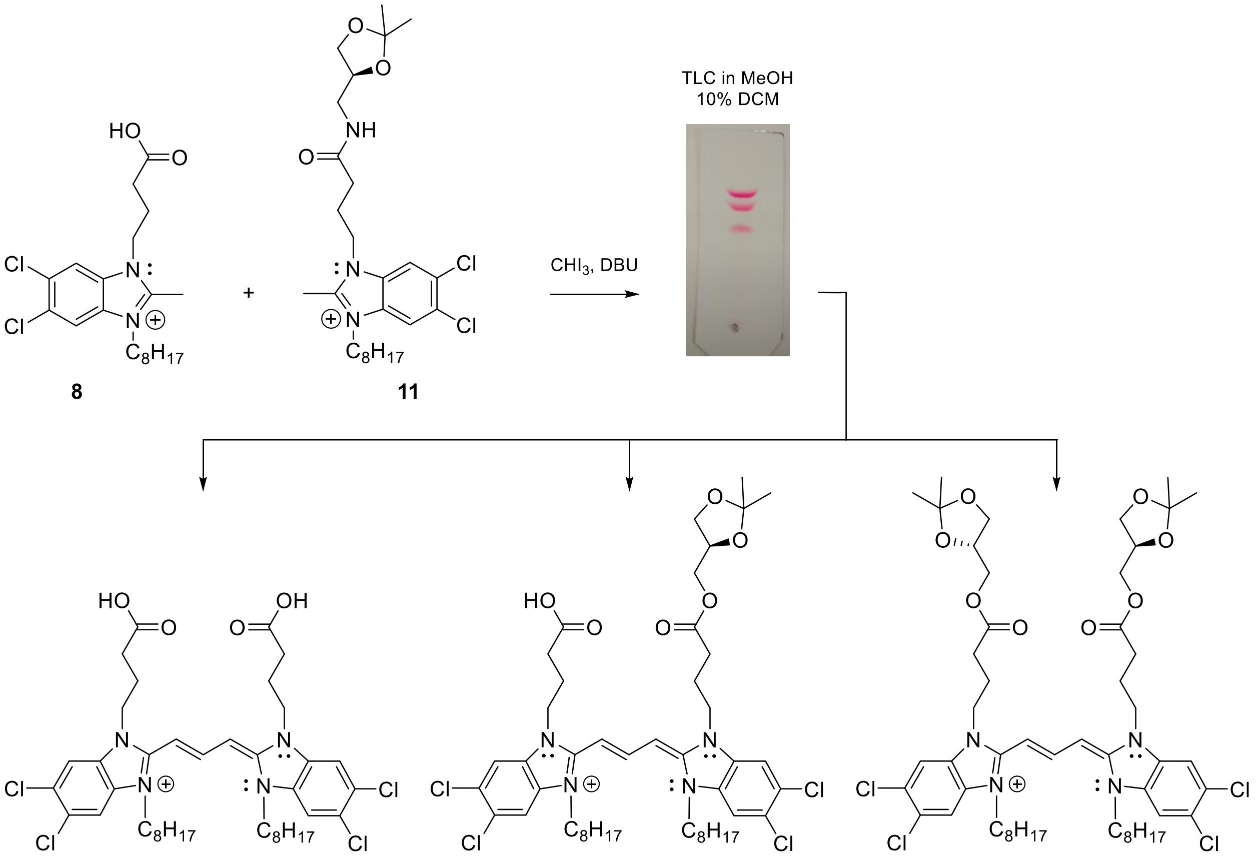

Scheme SI 3: Compound mixtures after the coupling of benzimidazoles 8 and 11 on the reaction pathway to achiral mesomer (1c).

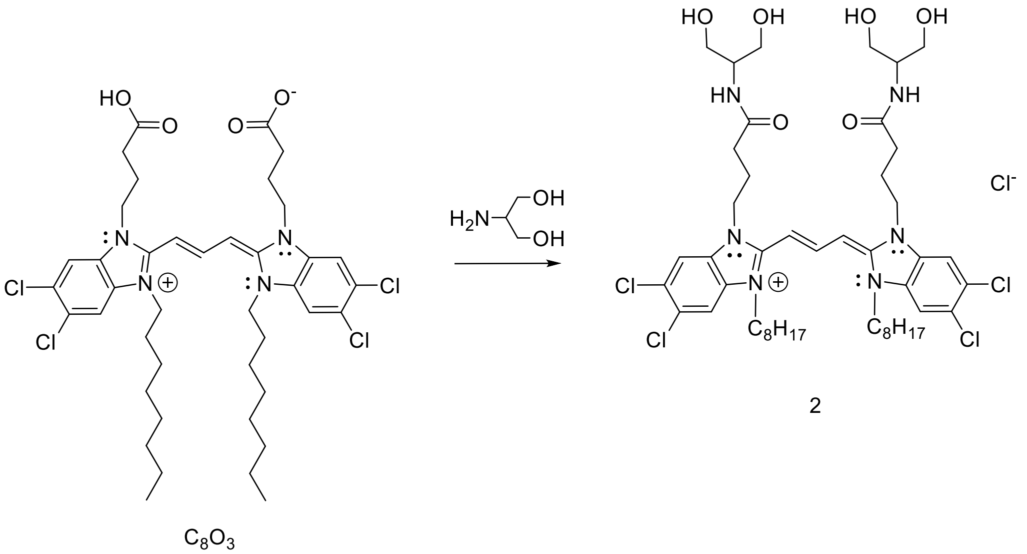

Scheme SI 4: Synthesis of the conformer (2); HATU, DMF, DIPEA, r.t., 2 h.

Spectroscopy

Molar extinctions

Table SI 1 Molar extinction coefficients of 0.1mM molecular solutions of the amino-propanediol dyes in methanol and DMSO.

	1a	1b	1c	2
**MeOH**	112,000	105,000	115,000	125,000
**DMSO**	121,000	97,000	89,000	147,000

Structural characterization

Molecular dimensions

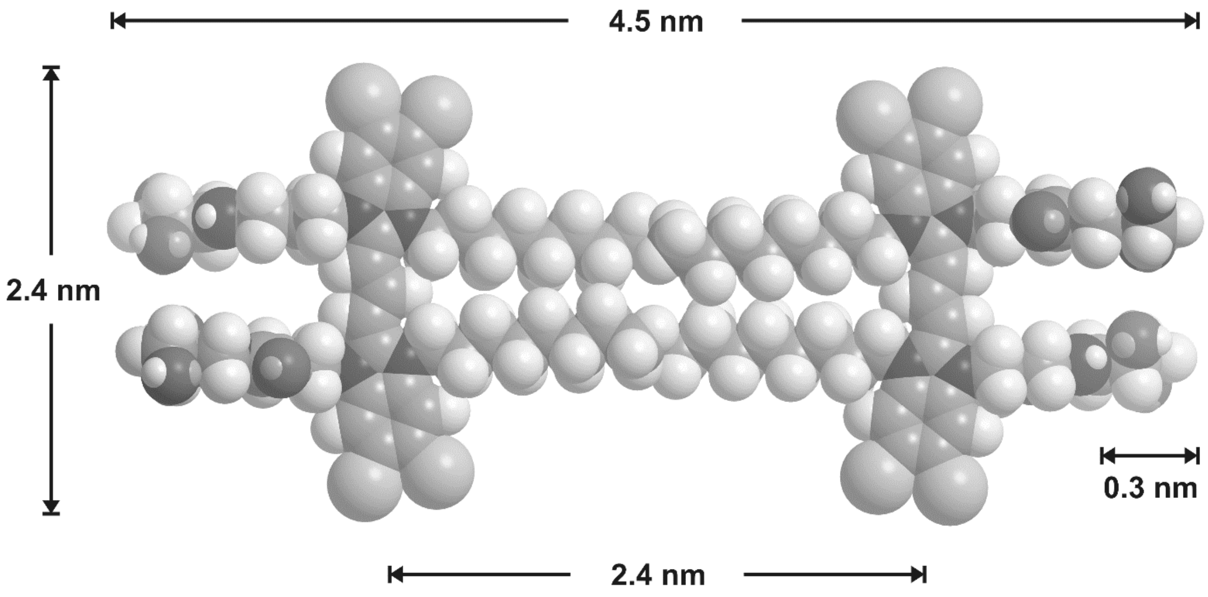

Figure SI 1: Molecular model of a pair of the amino-propanediol dyes forming the bilayer membranes and the tube walls in a typical tail-to-tail arrangement. The resulting overall length of the model as well as the distance between the opposite chromophores fit well with the measures of the line plots of the doublelayered tubes and the heights of the sheets as determined by AFM.

Mesomer in water

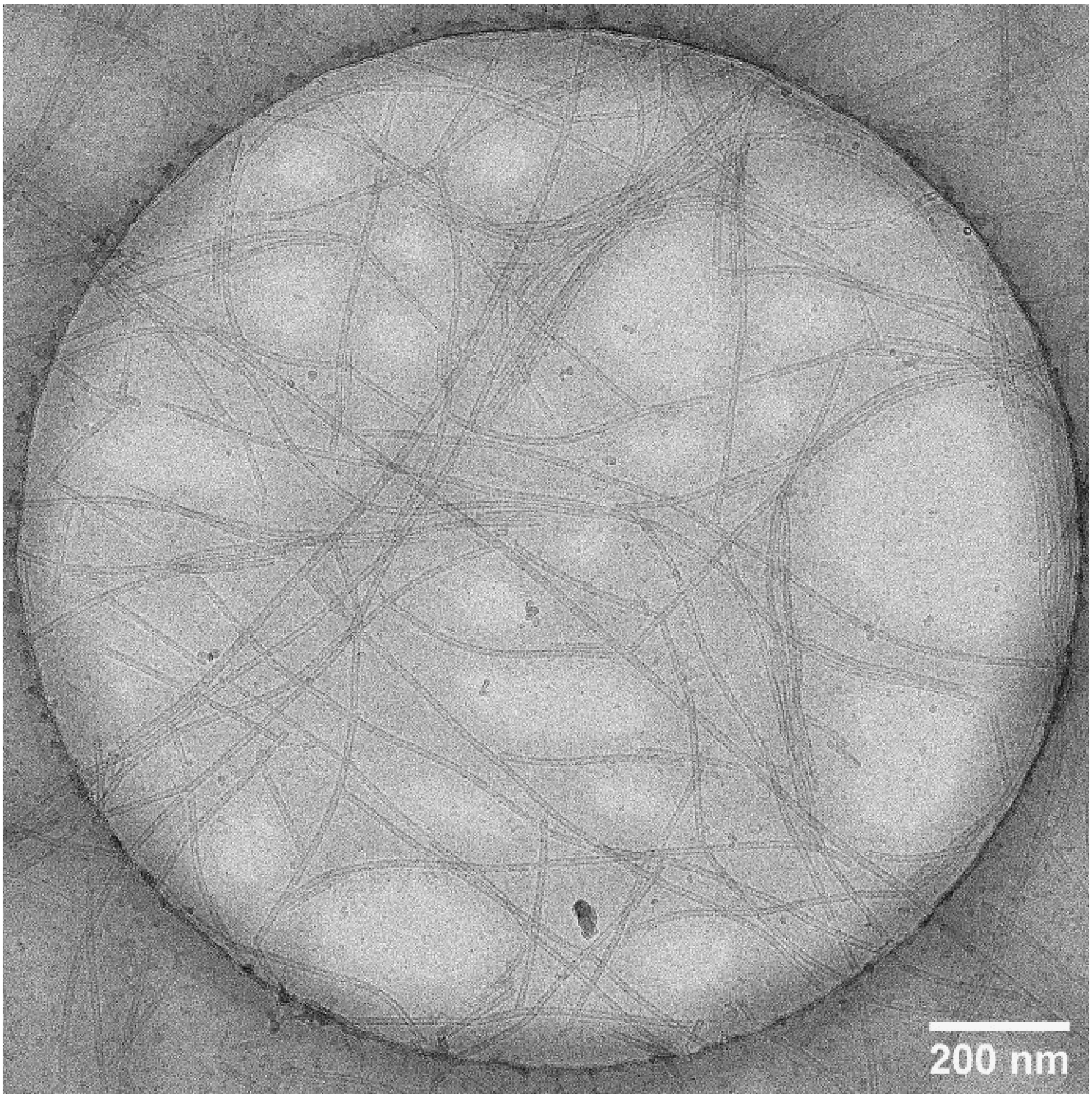

Figure SI 2: Cryo-TEM micrograph of a 21 days old mesomer (**1c**) solution in water discloses that once formed individual tubes remain detached even after long storage times.

Upon long storage times (up to 186 days) the composition of a particular meso dye solution did not change, i.e. once formed the long individual tubes remain detached as shown in Fig. SI 2 and do not bundle.

Mixture of enantiomers

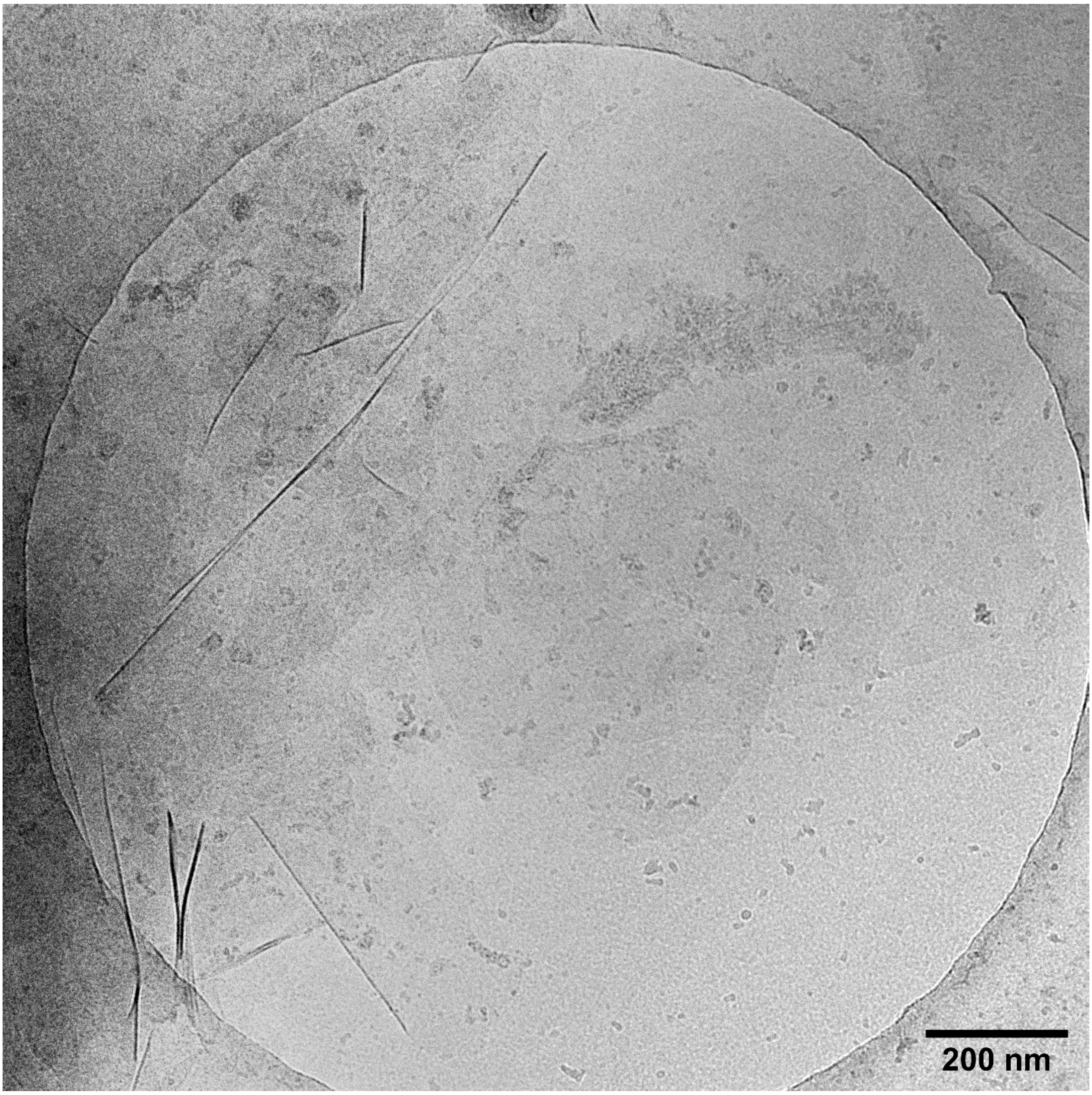

Figure SI 3 By first mixing both enantiomers (1a and 1b) and then dissolving them in pure water similar sheets like those observed from the pure enantiomers are formed. Beside the typical folds there are no indications of curvature or even tube formation.

To test whether the mixture of the contrary configured enantiomers forms tubes we dissolved the pure enantiomers (**1a** and **1b**) in one-to-one ratio in water. UV/vis spectra of this mixture displayed only one narrow band at 584 nm, just like the pure enantiomers. In cryo-TEM measurements, only sheet-like aggregates were found (Fig. SI 3) and no tendency of buckling or even tube formation was observed. The mixing experiments prove that only the mesomer (**1c**) alone induces curvature to form tubes and tube bundles.

Cryo-electron tomography (cryo-ET) of the mesomer

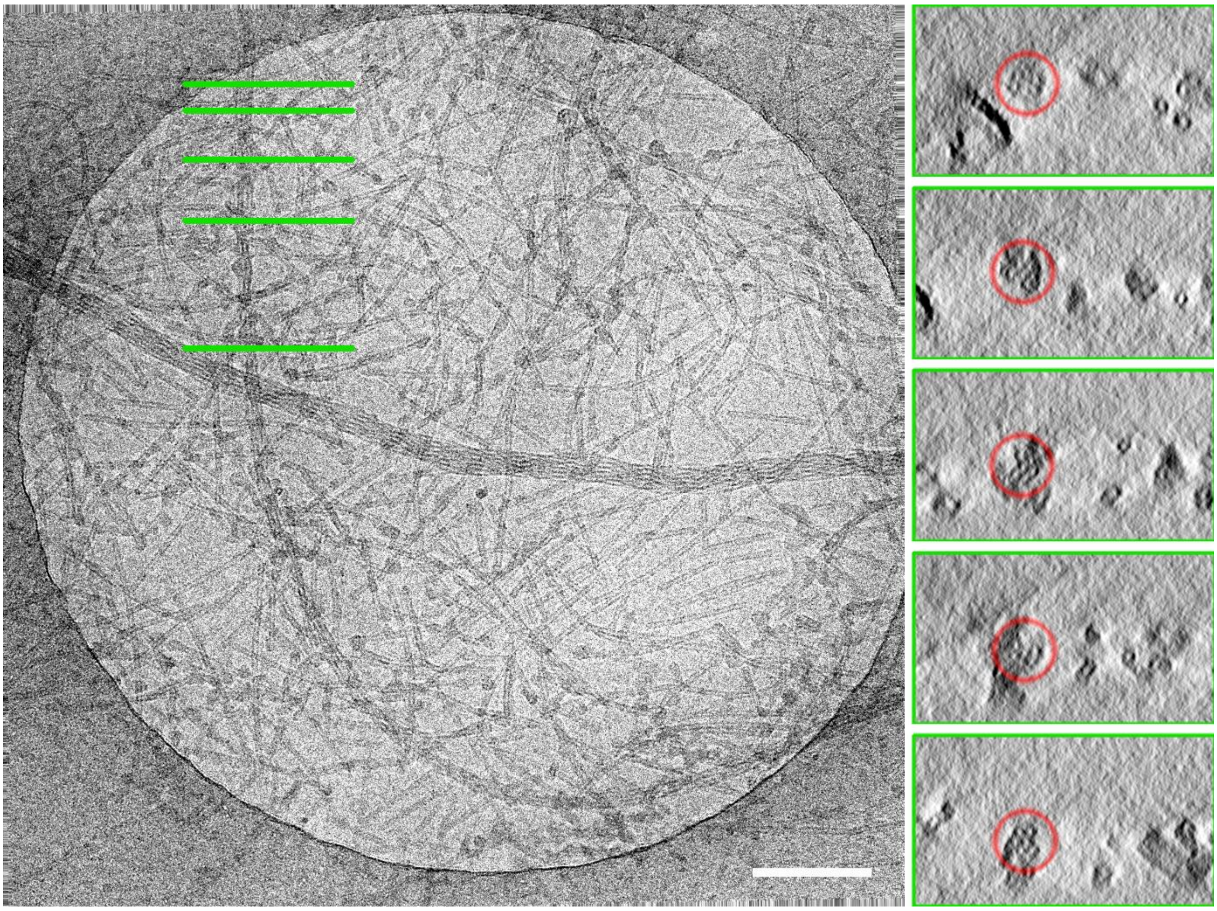

Figure SI 4: Slices of the tomogram disclose the twisted bundles' organisation from six tubes (right). The approximate locations of the displayed slices are indicated by green lines in the micrograph (left). Bar represents 200 nm. By following the bundle downwards from the top, the rotation of the bundle cross-section and thus the twist of the bundle becomes obvious. See the movie, also.

Modelling of the tube bundles

Geometrical considerations

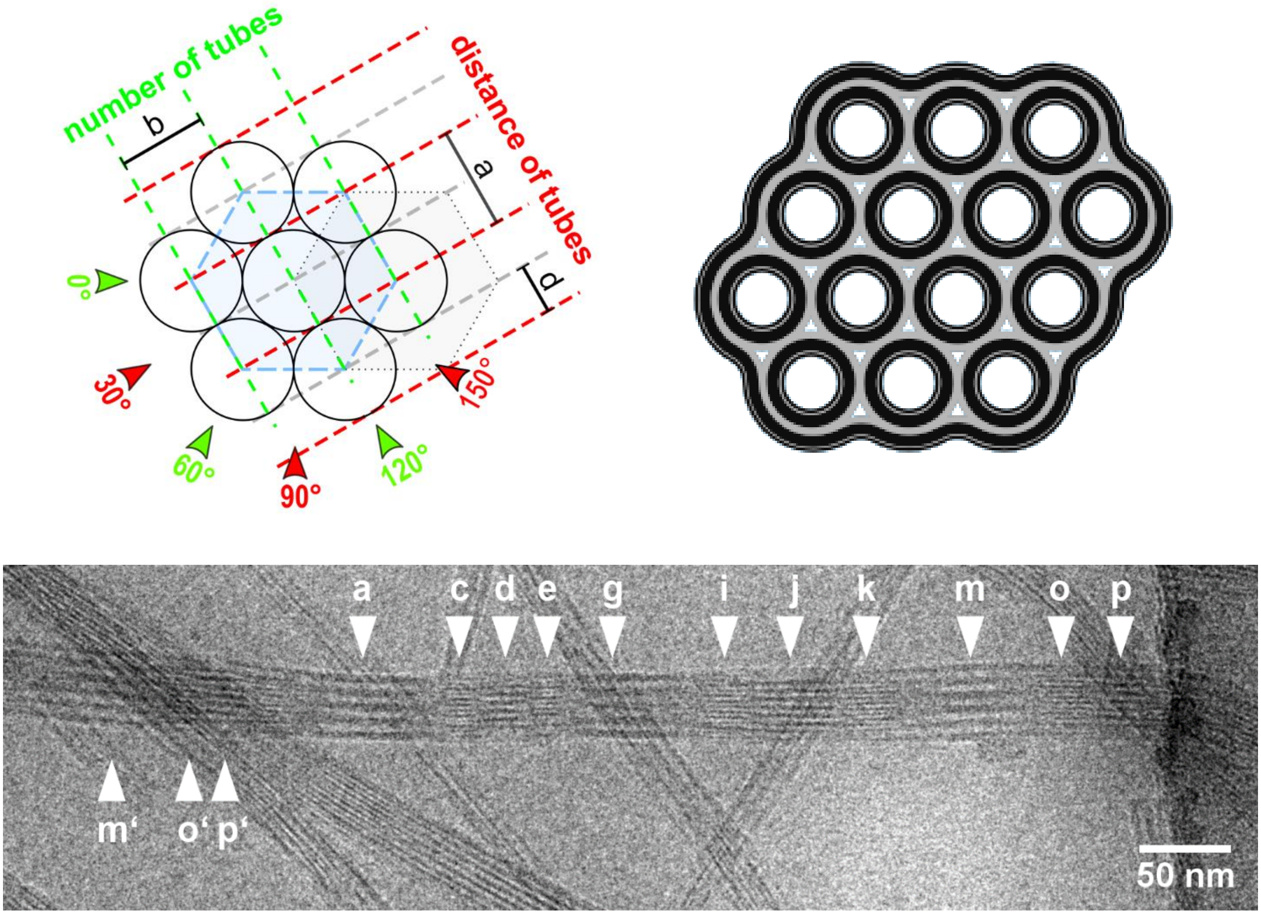

Figure SI 5 Geometrical dependencies of hexagonal arranged cylinders help to find structural parameters like tube distance (red dotted lines) as well as the number of tubes (green dotted lines) directly from the TEM micrographs (bottom). With these parameters the underlying cross-section (density maps) of the bundle (right) can be constructed and used to generate a 3D model of the respective aggregate. In the original data (bottom) the multiplicities are 4, 4, and 5 at positions a, g, and m and the tube diameter is 8.4 nm, i.e. double the line spacing in positions d, j, and p.

The geometrical parameters for the bundle model are defined in Fig. SI 5 left. A line pattern with a repeating distance b is obtained in projections at rotation angles of 0°, 60°, 120°. The multiplicity of this pattern reflects the number of tube layers. Of course, different values are possible for the three different rotation angles and allow to describe the exact number and arrangement of constituting tubes of the bundle. At 30°, 90°, 150°, a line pattern with a second repeat distance $d = a/2$ is observed, where a is the lattice constant of the hexagonal lattice which is equal to the tube's diameter. Parameters b and a are related by:

$$\mathrm{b} = d\frac{\sqrt{3}}{2} = 2\mathrm{a}\frac{\sqrt{3}}{2} = \mathrm{a}\sqrt{3} \approx 1.732\ \mathrm{a} \qquad (1)$$

Based on these considerations we are able to identify the basic parameters of each of the tube bundles displayed in the micrographs from only two parameters: (1) the multiplicities of the widest line sequences at 0°, 60°, and 120° mark the number of tube layers, (2) the twofold line distance (a) of the second widest sequence at 30°, 90°, and 150° denotes the nearest spacing of the tubes, i.e. their diameters. On a twisted bundle a specific line pattern should repeat along the axis every third time the pattern appears. For the model tube (Fig. SI 5) the multiplicities are 4, 4, and 5, i.e. the tube layers in the designated directions (a, g, and m) and a diameter of 8.4 nm (d, j, and p).

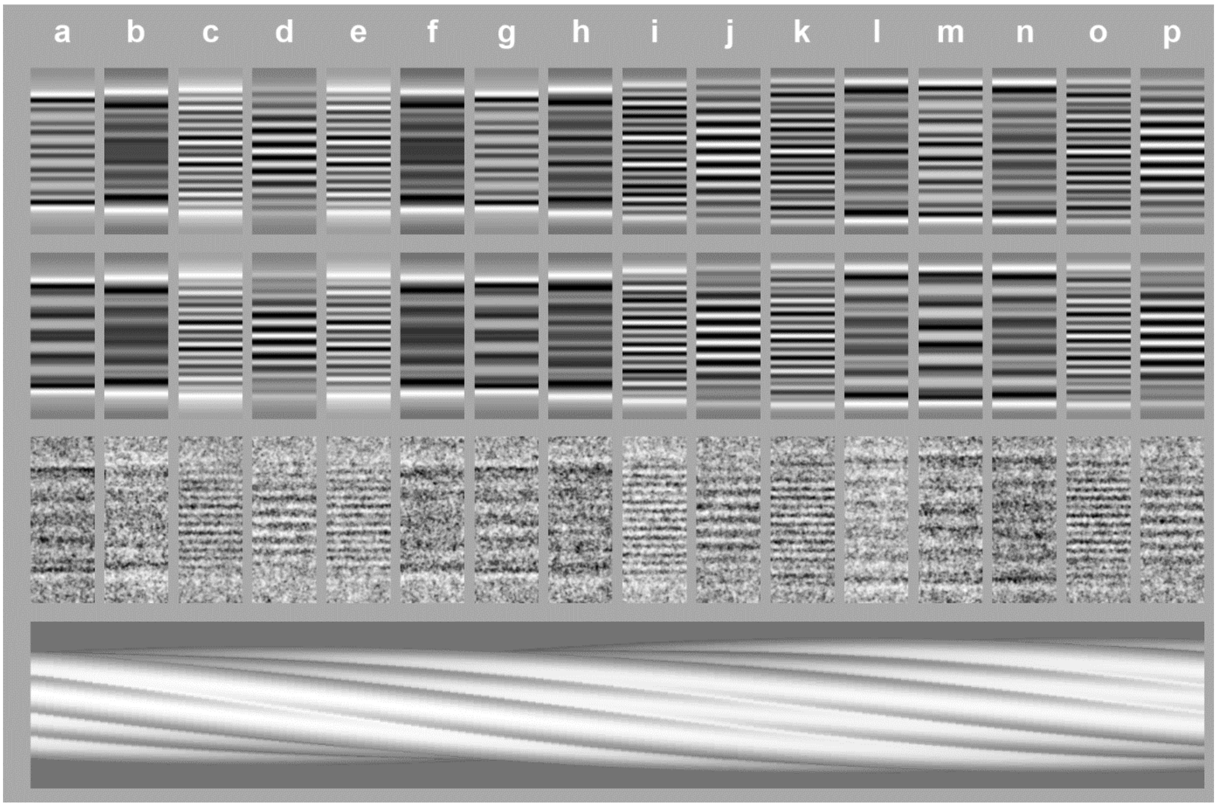

Figure SI 6: Enlarged view of the back projections displayed in the main text. (Row 1) side-by-side arrangement of the tubes. (Row 2) compressed arrangement of the tubes with reduced voids at the triangular contact faces. (Row 3) Original data from the model tube in displayed in Fig. SI 5 (bottom). The last row depicts a twisted surface view of the 3D model used to calculate the back projections in rows 1 and 2. The surface is aligned with the back projections above.

For a consistent simulation, which reproduces the experimental pattern as closely as possible, we have to discuss different molecular packing scenarios for the constituent tubes. A *normal* double-layer construction of the tube walls is excluded from the density profile of the cross-section of the bundle as obtained from cryo-ET (see main text). Inverted monolayer arrangements are the geometric alternative, which would, however, expose the hydrophobic alkyl chains outwards. Such orientation enables hydrophobic interactions between several tubes. Upon bundling, they can diminish their contact with the aqueous surrounding. The outermost surface of the bundles, however, remains exposed to water. A problem, that can in principle be overcome by postulating an additional monolayer, which envelopes the entire bundle with its polar head groups exposed to the solvent, thus shielding the inner hydrophobic bundle as realized in Fig. SI 4 right. Such type

of bundle construction was proposed before for C8S3 by Eisele *et al*. from their analysis of cryo-ET data.[1]

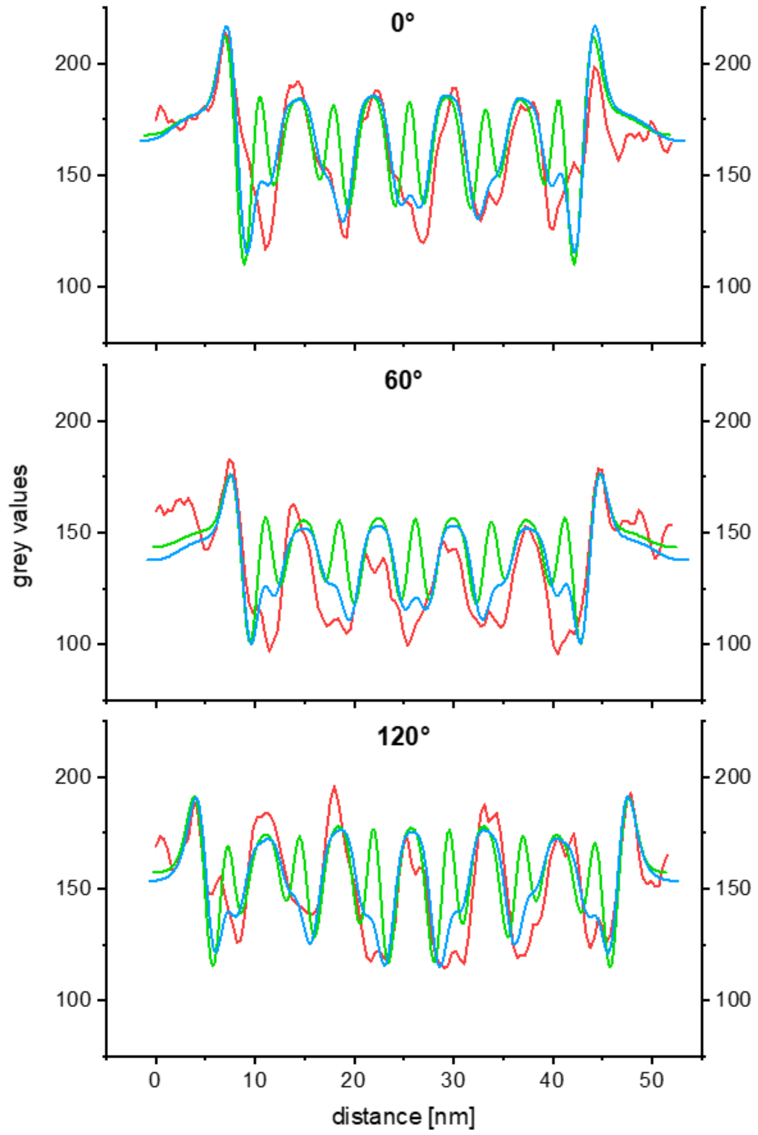

Figure SI 7: Comparison of line plots of the back projections at 0° (a), 60° (g), and 120° (m) in Fig. SI 6 (rows 1 and 2) with the original data from the micrograph at respective positions (Fig. SI 5 bottom and Fig. SI 6 row 3). The graphs reveal distinct differences between the two models, i.e. the *side-by-side* configuration (green) and the compressed configuration (blue) and indicate a better consistency of the latter with the original data (red).

References

[2] D. M. Eisele, D. H. Arias, X. Fu, E. A. Bloemsma, C. P. Steiner, R. A. Jensen, P. Rebentrost, H. Eisele, A. Tokmakoff, S. Lloyd, K. A. Nelson, D. Nicastro, J. Knoester, M. G. Bawendi, *PNAS* **2014**, *111*, E3367-E3375.
